# A novel sub-differentiable hausdorff loss combined with BCE for MRI brain tumor segmentation using UNet variants

**DOI:** 10.1038/s41598-025-33136-x

**Published:** 2025-12-22

**Authors:** Gurugubelli Visalakshi, Laavanya Mohan

**Affiliations:** Vignan’s Foundation for Science, Technology and Research, Guntur, Andhra Pradesh India

**Keywords:** Sub-differentiable hausdorff loss (SDHL), Tumor segmentation, Boundary alignment, Loss function design, Gradient-based optimization, Medical image analysis, Cancer, Computational biology and bioinformatics, Engineering, Mathematics and computing, Medical research, Oncology

## Abstract

Brain tumor segmentation is a crucial yet complex task in medical image analysis, playing a vital role in accurate diagnosis and treatment planning. However, it faces several inherent challenges: (1) class imbalance, where tumors occupy a minimal portion of MRI scans, leading models to favor healthy tissue; (2) low sensitivity to small or irregular tumors, resulting in high false negatives; and (3) limitations of standard loss functions, which often fail to properly emphasize tumor boundaries.TraditionalHausdorff Loss, despite its effectiveness in boundary alignment, suffers from non-differentiability and high sensitivity to outliers, leading to unstable optimization and poor boundary predictions. To address these challenges, we propose a novel Sub-Differentiable Hausdorff Loss (SDHL), which introduces a smooth, differentiable formulation enabling stable gradient-based learning and robust boundary alignment. Furthermore, to balance fine boundary precision with accurate tumor region segmentation, we introduce a novel SDHL combined with BCE loss. This combination leverages SDHL’s boundary sensitivity and BCE’s region accuracy, resulting in sharper tumor boundaries and more complete segmentation outcomes. This study presents a deep learning-based brain tumor segmentation pipeline utilizing various UNet-derived architectures including UNet, UNet+, VNet, UNet++, and Attention UNet, all trained using the proposed loss functions. The models evaluated using performance metrics such as Accuracy, Precision, Recall, Dice Score, and Intersection over Union (IoU). Among the tested configurations, Semantic segmentationmetricsofAttention UNet with SDHL + BCE outperformed all others, achieving 99.71% accuracy, 91.06% precision, 91.85% recall, 90.16% Dice score, and 80.78% IoU. The integration of the proposed loss functions significantly improves segmentation quality, addressing key challenges and offering a robust solution for brain tumor segmentation in medical imaging.Additionally, the Attention UNet model demonstrated strong consistency across evaluation metrics, achieving 97.92% accuracy, 98.76% precision, 98.05% recall, 98.57% F1-score, and a Matthews correlation coefficient of 93.41%, indicating robust and reliable performance.

## Introduction

 In the brain or its surrounding structures, brain tumors are aberrant growths or unchecked cell proliferation. The brain is the main organ of the central nervous system (CNS), and it is essential for processing, integrating, and coordinating several body processes, as well as for decision-making and signal transmission throughout the body. The brain’s extremely complex anatomical structure makes it susceptible to a wide range of neurological conditions, such as infections, multiple sclerosis, strokes, dementia, developmental abnormalities, brain tumors, traumatic brain injuries, and chronic headaches^1^. Brain tumors are regarded as one of the most aggressive and difficult forms of cancer among these conditions. Brain tumors are divided into two main groups by the World Health Organization (WHO). (1) High-grade gliomas (HGG) are malignant tumors that grow aggressively and recur frequently. They are derived from glial cells. (2) Low-grade gliomas (LGG) are tumors that grow more slowly but have the potential to eventually develop into higher-grade cancers.

In order to limit brain damage and prevent tumor growth, early detection and prompt medical intervention are essential. One of the most popular imaging methods for identifying brain tumors is magnetic resonance imaging (MRI). It is a vital tool for tumor detection and analysis due to its capacity to provide multi-dimensional, high-resolution pictures with superior soft tissue contrast^2^. When it comes to detecting brain tumors, Fluid-Attenuated Inversion Recovery (FLAIR) imaging is one of the most important MRI modalities. FLAIR makes aberrant tissues more visible by suppressing normal cerebrospinal fluid (CSF) signals. This renders it especially beneficial for identifying diffuse brain cancers and low-grade gliomas (LGG)^3^. According to recent research, FLAIR sequences are crucial for tumor segmentation tasks because they can effectively differentiate between healthy and cancerous brain tissue. Segmentation algorithms must be strong enough to manage heterogeneous data, though, because tumor appearance varies among patients and MRI environments. Inter-observer variability results from the time-consuming and human error-prone nature of manual tumor segmentation. The need for automated and accurate segmentation techniques created especially for FLAIR MRI images is therefore increasing^4^.

**Fig. 1:**
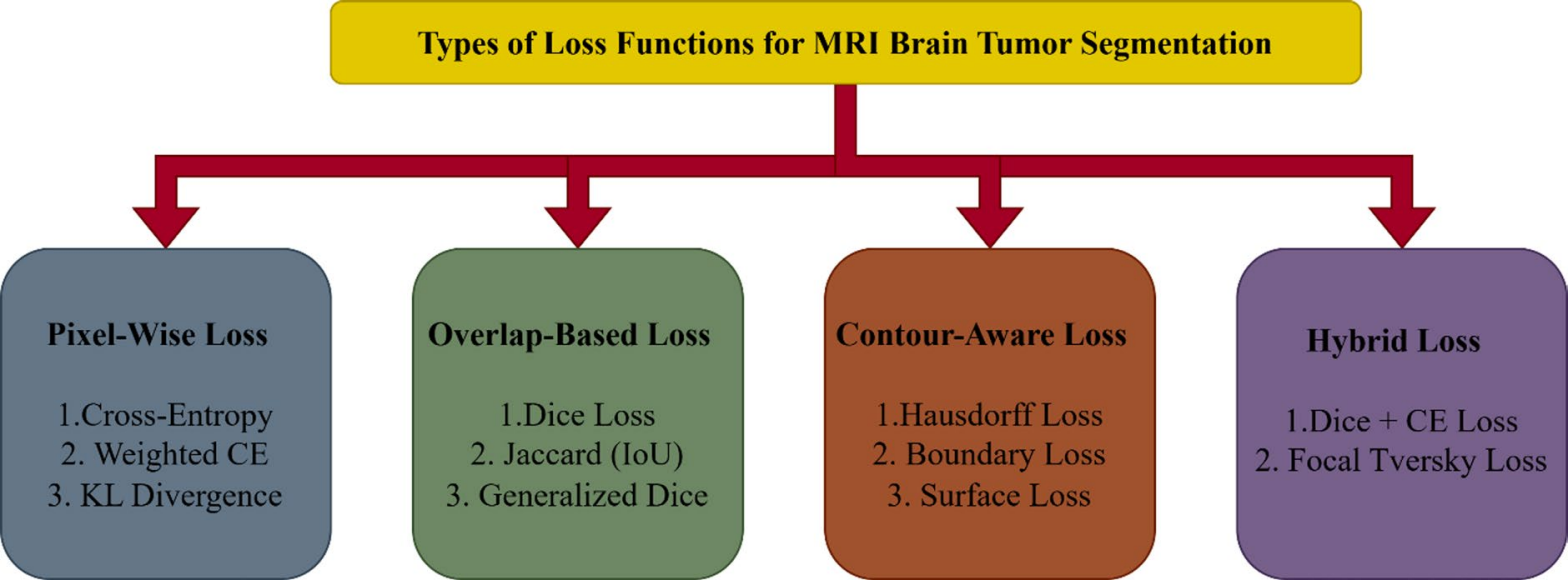
Types of loss functions.

Various loss functions used for MRI brain tumor semantic segmentation into four primary groups: Pixel-Wise Loss, Overlap-Based Loss, Contour-Aware Loss, and Hybrid Loss as shown in Fig. 1. Each category addresses specific challenges in medical image segmentation and contributes uniquely to improving model accuracy and reliability.Pixel-Wise Losses evaluate the discrepancy between predicted and actual labels at the individual pixel level. Common examples include Cross-Entropy (CE) Loss, Weighted Cross-Entropy (WCE) Loss, and Kullback-Leibler (KL) Divergence Loss. These losses are particularly effective when dealing with balanced datasets and aim to maximize per-pixel classification accuracy. However, they may struggle with highly imbalanced data, which is typical in brain tumor segmentation, where tumors often occupy a very small portion of the image.

Overlap-Based Losses focus on maximizing the similarity between predicted and ground truth segmentations. Examples include Dice Loss, Jaccard (IoU) Loss, and Generalized Dice Loss (GDL). These losses are well-suited for imbalanced data and small object detection, as they directly optimize for overlap metrics used in medical evaluations. They help the model learn better representations for tumor regions that occupy fewer pixels in an image.Contour-Aware Losses are designed to enhance the precision of boundary delineation, which is crucial for clinical applications such as surgical planning and radiation therapy. Examples include Hausdorff Distance (HD) Loss, Boundary Loss, and Surface Loss. These losses pay special attention to the borders of tumors, reducing over-segmentation or under-segmentation at the edges and improving spatial accuracy.Hybrid Losses combine the advantages of multiple loss types to provide a more robust and balanced training signal. For instance, Dice + Cross-Entropy Loss leverages both pixel-wise accuracy and region-based overlap, while Focal Tversky Loss (FTL) is particularly useful for detecting small and hard-to-segment tumors by focusing on difficult samples. The choice of loss function plays a critical role in the success of brain tumor segmentation models.

To establish the foundation for the proposed research, the following section reviews recent developments in brain tumor segmentation and highlights the limitations of existing loss functions used in medical image analysis.

## Literature survey

Segmentation accuracy has greatly increased because to deep learning-based segmentation models such Transformer-based architectures^5^, UNeXt with Wave-MLP^6^, and SEResU-Net^7^. However, the selection of loss functions, which maximize network training by reducing segmentation mistakes, has a significant impact on these models’ performance. In brain tumor segmentation, where tumors make up a very small portion of the MRI picture, pixel-wise loss functions like CE Loss and WCE Loss enhance segmentation at the pixel level but frequently fall short in addressing the severe class imbalance. Dice Loss and Jaccard (IoU) Loss are examples of overlap-based loss functions that maximize region-level overlap to enhance segmentation. Small or irregularly shaped tumors are frequently misclassified because of their difficulties with fine-grained boundary delineation^8,9^.

Boundary Loss is one of the contour-aware loss functions that have been introduced to improve boundary precision. To increase segmentation accuracy for irregular tumor formations, these losses concentrate on reducing the discrepancy between expected and actual tumor borders^10,11^. Nevertheless, these techniques continue to suffer from training instability and computational inefficiency when used on intricate medical images. The goal of hybrid loss functions, like Focal Tversky Loss (FTL) and Dice + Cross-Entropy Loss, is to combine the advantages of several losses. Even though these techniques increase generalization, they still have trouble segmenting tumors with intricate forms and datasets that are extremely unbalanced^12,13^.

Despite being useful for preserving boundaries, traditional Hausdorff Distance Loss is computationally costly and non-differentiable, which restricts its applicability to contemporary deep learning optimizers^14^. In order to overcome these drawbacks, we create a new loss function called the Sub-Differentiable Hausdorff Distance Loss Function, which maintains HD Loss’s boundary-sensitive characteristics while guaranteeing steady model training through smooth gradient propagation.Several studies focus on designing robust loss functions to enhance segmentation performance: Jain et al. (2023) introduced PIXIE, a novel loss function for binary semantic segmentation, aimed at improving boundary preservation and handling class imbalance effectively^15^.Kim et al. (2023) proposed a Bidirectional Meta-Kronecker Factored Optimizer combined with Hausdorff Distance Loss for few-shot medical image segmentation, enhancing generalization in low-data scenarios^16^.Islam et al. (2024) presented Adaptive Focal Loss, which dynamically adjusts focus on hard-to-classify pixels, improving segmentation accuracy in complex regions^17^.Malhotra et al. (2022) introduced a compound-based loss function for glioma segmentation, balancing pixel-wise and structural accuracy using deep learning techniques^18^.

Recent research has explored transformer models and attention mechanisms for better feature extraction: Cao et al. (2023) developed Swin-Unet, a transformer-based UNet model for medical image segmentation, leveraging hierarchical feature representation to capture both local and global context^19^.Mazumdar& Mukherjee (2022) proposed Efficient Spatial Attention Convolutional Networks (ESACN), which use composite loss functions to improve automatic MRI brain tumor segmentation^20^.Allah et al. (2023) introduced Edge U-Net, an improved deep learning model incorporating boundary information for enhanced tumor segmentation accuracy^21^.

Hybrid models and ensemble methods have been explored to achieve superior performance: Jin et al. (2023) designed a multi-branch segmentation network that combines local and global information synchronous learning to refine tumor segmentation boundaries^22^.Roshan et al. (2024) developed a deep ensemble model with a novel sampling method and loss function, significantly improving segmentation robustness^23^.Magadza&Viriri (2021) provided an extensive survey on deep learning techniques for brain tumor segmentation, covering traditional CNNs, UNet-based models, and GAN-based methods^24^.

**Table 1:** Existing works on brain tumor segmentation.

Reference	Dataset Used	Architecture	Loss Function	Focus	Performance Metrics	Key Findings
Iriawan, N et al. (2024)^8^	BraTS 2020	YOLO-UNet	Binary Cross-entropy Loss	Probability distribution	Correct classifcation ratio (CCR) is high CCR of about 97%.	BCE loss struggles with tiny lesion segmentation in unbalanced datasets and prefers majority class learning.
Ali, A et al. (2024)^10^	Br35H MRI image	PG-OneShot learning CNN	cross-entropy loss	Probability distribution	Dice Coefficient: (98.49%) IoU: (93.19%), Accuracy: (95.20%), Precision: (92.35%), Recall: (95.72%).	Affects uncommon tumor segmentation by prioritizing the majority class and struggling with invisible tumor variations.
Mohammed, Y et al. (2025)^12^	BraTS 2019	ERU-Net	weighted dice loss and Weighted Cross-Entropy Loss	Focus on minority class.	dice coefficient of TC:0.881, ET: 0.882, and WT: 0.891.	Due to its dependence on the distribution of training data and feature representation constraints, ERU-Net enhances segmentation but suffers from class imbalance and invisible tumor variations.
Hou, Cet.al. (2023)^9^	Lumbar MRI	FC-DenseNet	Gaussian Divergence Loss (Dvg-loss)	Probability divergence	Accuracy: 99.34, IOU 84.57.	With intricate parameter tuning for Gaussian scale selection, it is sensitive to noise and artifacts.
Ottom, Met.al. (2022)^11^	Low-grade Glioma	Znet Combines OfEncoderdecoder And Skip Connections	Dice Loss	Region overlap	DSC: 0.96 during model training and DSC: 0.92. Pixel accuracy: 0.996, F1 score: 0.81. MCC: 0.81.	Evaluation of pixel accuracy might not be a good way to assess semantic segmentation when there is class imbalance in the segmentation of MR images.
Cahyo, Get.al. (2023)^25^	A Public Dataset Of 330 Images	Mobilenetv2 + u-net Architectural	Gaussian And Poisson loss	Probability divergence	Accuracy:99.47% (High valdata) Accuracy:98.99% (Low val data) gaussian noise:99.64% poisson noise:99.5% Highdice-score: 82.80% Low dice-score 69.18% gaussian noise:87.83% poisson noise: 83.10%	Gaussian scale parameter tweaking adds complexity, and high noise makes feature extraction more difficult.
Kharaji, Met.al. (2024)^26^	BraTS (BraTS-GLI &BraTS-PED)	advanced nnU-Net	Hausdorff Distance Loss	Boundary accuracy	For BraTS-GLI DSC WT- 0.90, ET- 0.79, TC- 0.81. For BraTS-PED DSC WT- 0.85, ET- 0.60, TC- 0.69.	Advanced nnU-Net with residual blocks, attention gates, and Hausdorff distance loss improves segmentation but struggles with pediatric tumor cases.
Han, Yet.al.(2021) ^14^	liver; tumor	Cascaded 2.5D fully convolutional networks (FCNs)	Boundary Loss	Contour alignment	For Tumor Dice and IOU Scores: **74.5**,** 59.4**	While it is unable to fully utilize 3D spatial context, the 2.5D FCN with boundary loss enhances liver and tumor segmentation.
Celaya, Aet.al. (2023)^13^	LiTS and BraTS	nnUNet	Surface Loss	Distance from boundary	DSC for WT: 0.9087, TC: 0.8448, ET: 0.7587.	In unbalanced medical datasets, Generalized Surface Loss improves segmentation, decreases Hausdorff Distance and increases border precision.
Yan, Cet.al (2022)^7^	BraTS2018 and BraTS2019	SEResU-Net	Dice + Cross-Entropy Loss	Overlap + Class Prob.	DSC Score: WT- 0.9373, TC- 0.9108, ET- 0.8758.	Performance is impacted by class imbalance and high computational cost, however SE blocks improve feature selection.
Dhamija, Tet.al. (2023)^5^	MRI Dataset, Lungs Segmentation, Nuclei Segmentation, Skin Lesion Segmentation	Unet-based	BCE + Dice loss	Overlap + Class Prob.	Accuracy: 99.71 IOU: 0.9734 and DSC: 0.8934	Performance in transformers is limited by rigid patching, necessitating a dynamic and effective patching approach.
He, Yet.al. (2024)^6^	BraTS 2018 MRI and COVID-19 CT	UNeXt and Wave-MLP	Focal Tversky Loss	Focus on minority class	dice scores of the ET-0.933, WT-0.925, TC-0.918 respectively.	Sensitive to parameter tuning and dependent on precise area measurement, particularly in noisy data.

The comparative Table 1 highlights recent advancements in tumor segmentation using deep learning across various datasets, architectures, and loss functions. Traditional loss functions like Binary Cross-Entropy (BCE) and Cross-Entropy, as used by Iriawan et al. and Ali et al., focus on pixel-wise classification but often struggle with small or rare lesion segmentation due to class imbalance. To address this, researchers such as Mohammed et al. and Yan et al. adopted weighted or hybrid losses (e.g., Dice + Cross-Entropy) to emphasize minority regions. Boundary-aware losses, including Hausdorff Distance Loss, Boundary Loss, Surface Loss, and Focal Tversky Loss, have been effectively employed by Kharaji et al., Han et al., and Celaya et al. to enhance contour alignment and boundary precision, particularly in irregular tumor shapes. Meanwhile, divergence-based losses like Gaussian Divergence Loss, used by Hou et al. and Cahyo et al., improve robustness to noise but require complex parameter tuning. Architecturally, U-Net variants remain popular, often enhanced with modules like attention gates (e.g., SEResU-Net, nnU-Net) or lightweight encoders (e.g., MobileNetv2) for better feature extraction. Although models generally report high Dice scores (above 0.90 for whole tumor regions), performance tends to drop for enhancing tumor and tumor core due to class imbalance. Key challenges across studies include imbalance in data, noise sensitivity, and insufficient feature representation. Notably, the analysis reveals a growing preference for combining region-aware and boundary-aware loss functions—such as the proposed SDHL + BCE hybrid—to balance global segmentation accuracy and precise boundary localization, ultimately leading to more robust and clinically useful tumor segmentation models.

The foundation for modern brain tumor segmentation was established by Ranjbarzadeh et al. (2021), who introduced attention mechanisms into deep learning models for multi-modal MRI segmentation. Their work demonstrated that incorporating attention improves the model’s ability to distinguish between tumor and non-tumor regions, resulting in better localization and segmentation accuracy^27^. Building upon this, Bagherian Kasgari et al. (2023) proposed a segmentation framework using Zernike moments and an enhanced ant lion optimization algorithm integrated with convolutional neural networks (CNNs). Their approach achieved improved precision and robustness in MRI-based tumor segmentation^28^. In the same year, Zhang et al. (2023) presented a hybrid CNN–Transformer model for medical image segmentation, showcasing how combining convolutional and transformer layers enhances both local and global feature representation^29^. Further advancements were made by Ranjbarzadeh et al. (2023), who provided a comprehensive review summarizing the progress of artificial intelligence tools in brain tumor segmentation. Their work highlighted ongoing challenges such as data imbalance, explainability, and generalization across clinical datasets^30^.

In 2024, significant contributions expanded the scope and applicability of segmentation models. Ranjbarzadeh et al. (2024) performed a comparative analysis of BraTS and real-clinical MRI datasets, identifying the performance gap between public and clinical data and stressing the importance of developing models that generalize effectively across diverse domains^31^. Sarshar et al. (2024) enhanced brain MRI classification by combining VGG16 and ResNet50 architectures with Multi-Verse Optimization, illustrating the effectiveness of hybrid feature extraction methods^32^. Beyond traditional segmentation, Ranjbarzadeh et al. (2024) also proposed a blockchain-based decentralized collaboration framework to ensure secure and transparent medical image processing^33^. In a subsequent study, they investigated the influence of backbone selection in YOLOv8 models, revealing its critical role in improving tumor localization performance^34^. Additionally, combining DeepLabV3 with attention mechanisms yielded superior segmentation accuracy across BraTS 2020 and private clinical datasets^35^.

Architectural innovations continued with the introduction of a hybrid UNet and Vision Transformer model by Ranjbarzadeh et al. (2024), which effectively fused multi-scale features for accurate segmentation^36^. Their Ebtag framework further contributed to interpretability by integrating EfficientNetV2, attention mechanisms, and Grad-CAM visualizations^37^. Parallel research by Zhang et al. (2024) introduced the CI-UNet, which merged ConvNeXt with cross-dimensional attention for robust feature extraction^38^. Wen et al. (2024) also developed MRCM-UCTransNet, an accurate 3D segmentation model for cone-beam CT images, highlighting the adaptability of attention-based architectures beyond brain imaging^39^. Most recently, Safarpour et al. (2025) introduced a dual-phase segmentation framework using Gumbel-Softmax and cascaded Swin Transformers, which achieved high performance in multi-class brain tumor segmentation tasks^40^. Collectively, these works show the steady evolution of brain tumor segmentation techniques, from CNN-based models to hybrid architectures that incorporate attention and transformers. The revised literature review now clearly situates our proposed work within this ongoing progression, demonstrating how it builds upon and advances the state of the art in accurate, interpretable, and generalizable brain tumor segmentation.

In traditional boundary-based approaches such as Hausdorff Distance Loss (HDL) and Boundary Loss (BL), the key limitation lies in their sensitivity to outliers and directional imbalance. The standard HDL computes the maximum distance between predicted and ground truth boundaries, which can cause instability when minor contour deviations exist. To overcome this, several modified versions were proposed Modified Hausdorff Distance (MHD) and Boundary Focal Loss (BFL), which improve robustness but still fail to capture bidirectional boundary consistency across irregular tumor shapes. Likewise, Hybrid loss formulations combining BCE + Dice + Boundary Loss emphasize both region overlap and contour matching, but their weighting mechanisms often ignore directional distance variations along the tumor periphery. The proposed SDHL introduces a directional distance mapping mechanism, where both inward and outward boundary discrepancies are symmetrically penalized using a direction-weighted Hausdorff gradient formulation. This approach reduces contour bias and ensures smoother convergence at complex edges. Moreover, integrating SDHL with BCE balances boundary precision with region-level accuracy, effectively mitigating class imbalance in medical segmentation. Across all architectures (UNet, UNet++, and AttUNet), our proposed SDHL + BCE combination consistently achieves the highest IoU and Dice scores, outperforming other loss functions.

Challenges of the existing work are explained as follows:

1. Poor Boundary Precision: Most traditional loss functions (e.g., BCE, Dice) focus on region-level accuracy, leading to imprecise tumor boundary delineation.
2. Non-Differentiability of Hausdorff Loss: Traditional Hausdorff Loss, though boundary-sensitive, is not differentiable, making it unsuitable for gradient-based optimization in deep networks.
3. Sensitivity to Outliers: Hausdorff Loss heavily penalizes isolated segmentation errors, making models unstable and prone to poor convergence.

Building on the insights and limitations identified in the existing studies, the next section presents the proposed Sub-Differentiable Hausdorff Loss (SDHL) and its integration with U-Net variants for improved segmentation performance.

The contributions of the proposed work to overcome the above mentioned challenges are:

1. The proposed SDHL function effectively addressing its long-standing issues of non-differentiability and sensitivity to outliers in medical image segmentation.
2. By integrating SDHL with the Binary Cross-Entropy (BCE) loss, our method achieves a balanced improvement between boundary precision and regional accuracy, leading to more consistent and reliable segmentation outcomes compared to existing loss functions.
3. Our experimental evaluation across multiple U-Net variants (U-Net, VNet, UNet++, and Attention U-Net) consistently shows superior performance in terms of Dice Score, IoU, and Precision, demonstrating the practical effectiveness of the proposed work.

Building on the insights and limitations identified in the existing works, the next section presents the proposed Sub-Differentiable Hausdorff Loss (SDHL) and its integration with U-Net variants for improved segmentation performance.

## Proposed loss sunction

**Fig. 2:**
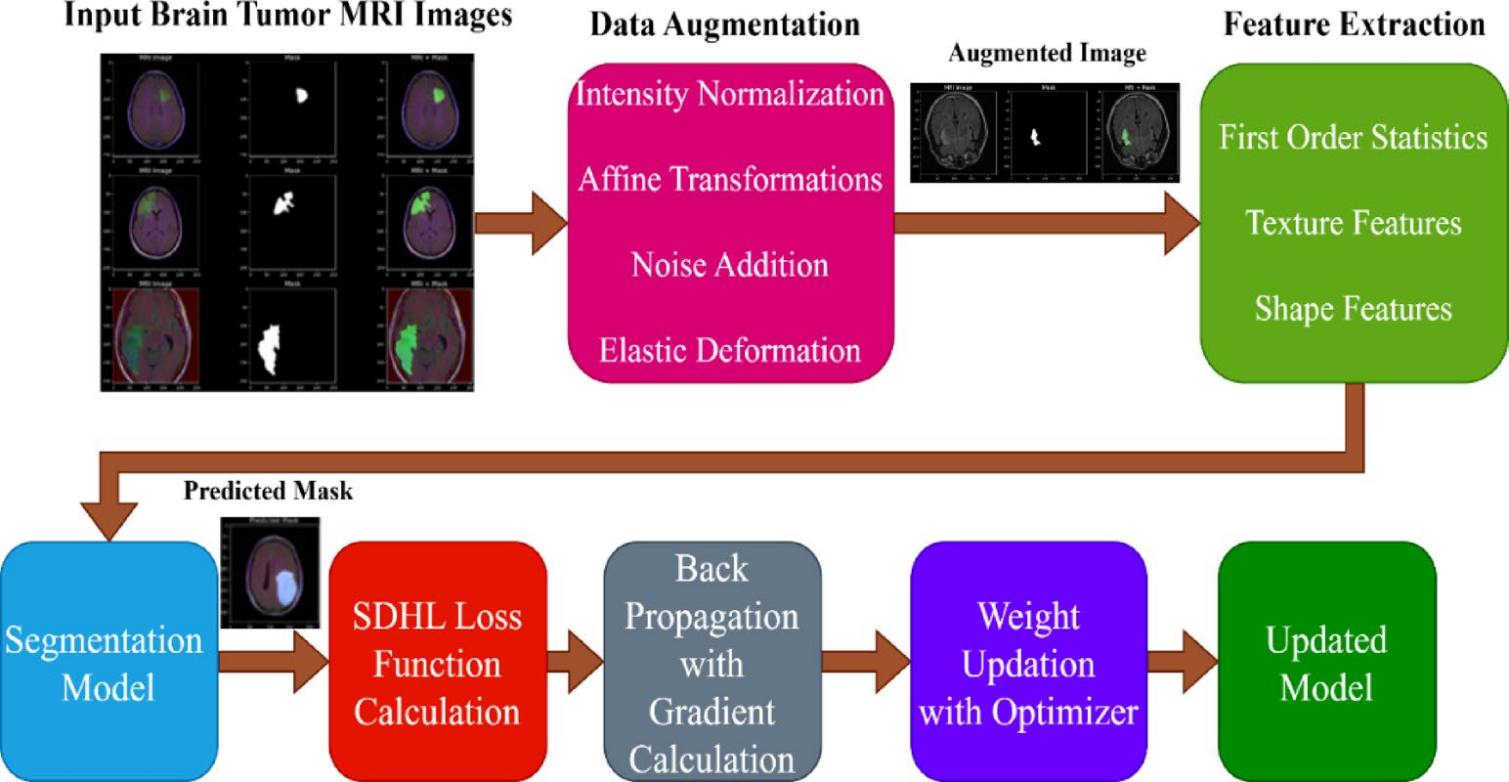
Working mechanism of novel loss function in brain tumor segmentation.

The working mechanism of Novel Loss Function in Brain Tumor Segmentation is shown in Fig. 2. The process begins with an MRI image, from which key features such as first-order statistics, shape features, and texture features are extracted. A segmentation model is then applied to generate a predicted tumor mask. To refine the accuracy of the segmentation, the predicted mask is compared with the ground truth using a Sub-Differentiable Hausdorff Distance Loss Function (SDHL), which measures the boundary discrepancy between the actual and predicted tumor regions. The computed loss undergoes backpropagation, where gradients are calculated and used to update the model parameters through an optimization algorithm. This iterative process results in an updated and improved model for more precise tumor segmentation. The entire workflow integrates feature extraction, deep learning-based segmentation, a novel loss function, and gradient-based optimization to enhance the accuracy of MRI-based tumor detection.

**Fig. 3:**
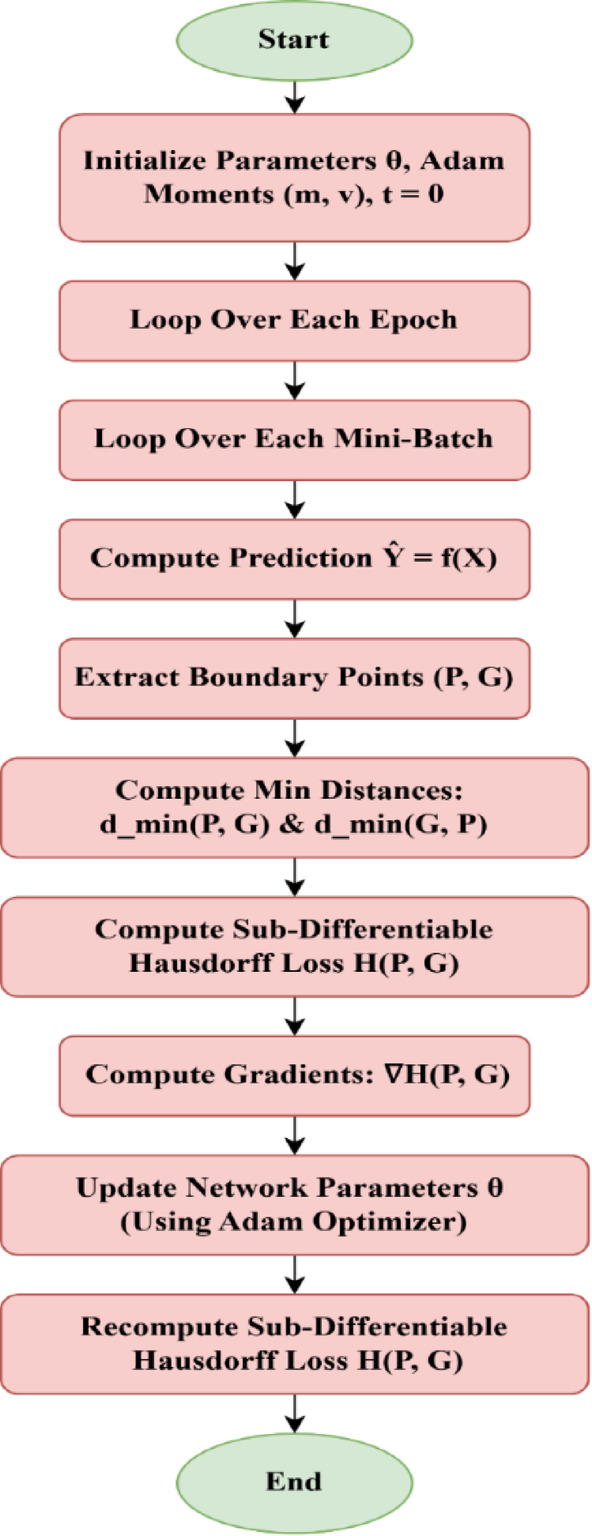
Flow chart for working of SDHL loss function.

The working of SDHL loss function is shown in Fig. 3. The process begins with the initialization of model parameters (θ) and Adam optimizer moments (m, v), with the time step set to zero. The model then iterates through multiple epochs, processing the dataset in mini-batches for computational efficiency. For each mini-batch, the model generates predictions (Y) from input data (X) and extracts boundary points from both the predicted (P) and ground truth (G) segmentations. The minimum distances between these boundaries are computed, followed by the calculation of the SDHL loss. Gradients of this loss function with respect to model parameters are then computed and used to update network parameters via the Adam optimizer. After each update, the SDHL loss is recomputed to assess improvements. This process continues until the specified number of epochs is reached, ensuring that the model learns to enhance segmentation accuracy by minimizing the boundary distance between predictions and ground truth.

The work flow of novel loss function shown in algorithm 1.

**Algorithm 1:**
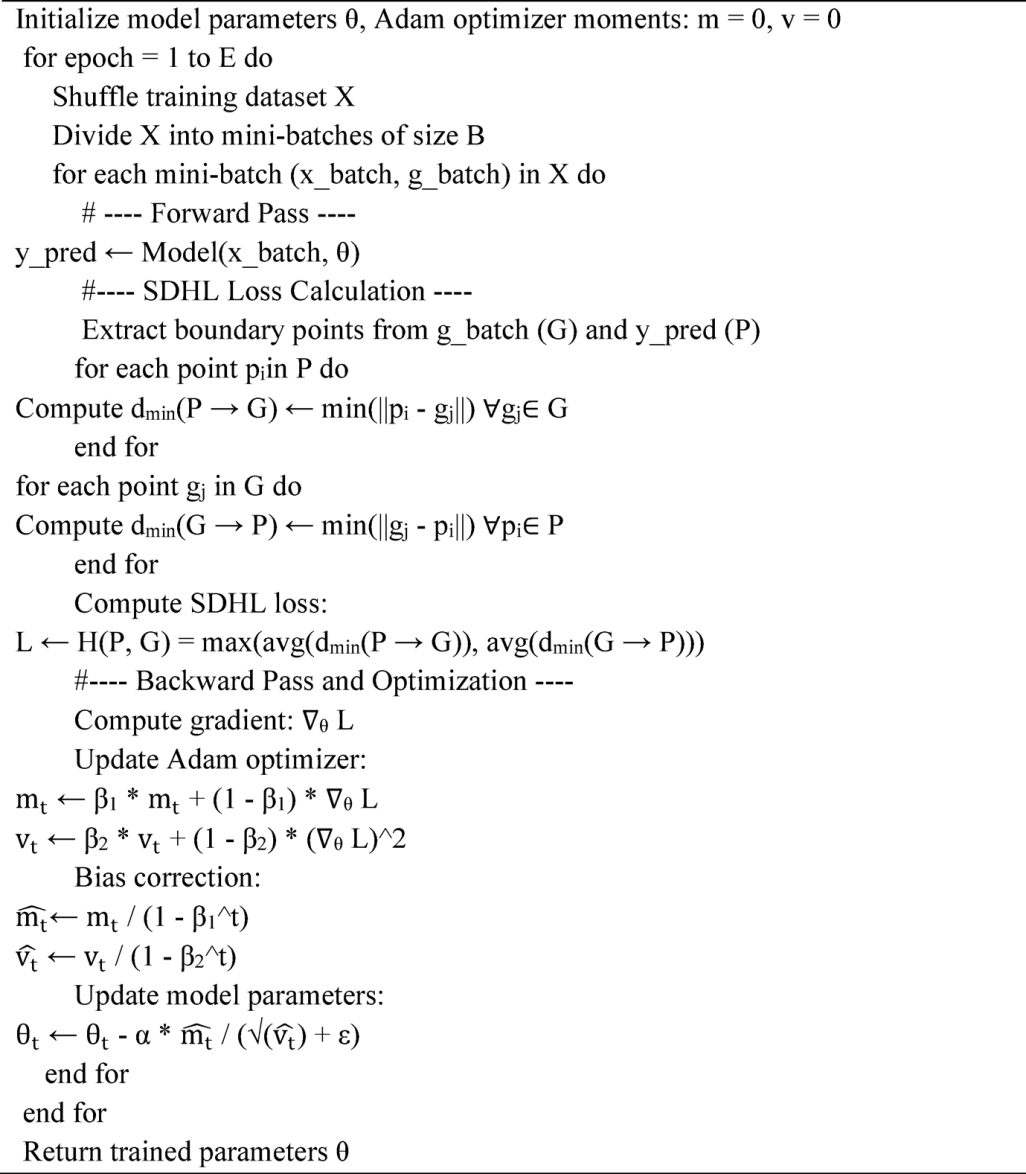
Working of novel loss function.

The step by step procedure and mathematical equations of the proposed loss function is explained as follows.

### Find the closest point

1. For each point in A(ground truth), find the closest point in B (prediction).
2. compute the minimum distance for every point in A.
3. Take the maximum of these minimum distances.

- **Repeat for B**
- For each point in B (prediction), find the closest point in A (ground truth).
- Compute the minimum distance for every point in B.
- Take the maximum of these minimum distances.

### Final hausdorff distance

- The maximum of these two values represents the worst-case deviation between prediction and ground truth.

1 $$\:{L}_{DHL}=\frac{1}{\left|P\right|}\sum\nolimits_{i}{d}_{min}\left({p}_{i},G\right)+\frac{1}{\left|G\right|}\sum\nolimits_{j}{d}_{min}\left({g}_{j},P\right)$$

The proposed **SDHL Loss** incorporates both pixel-wise classification accuracy and spatial distance constraints to improve tumor segmentation performance. Here’s how the loss function works in detail:

### Forward pass (computing the loss)

The loss function measures the spatial discrepancy between the predicted tumor mask and the ground truth. For each predicted point pi​ in the segmented mask, the algorithm computes the Euclidean distance to all ground truth points gj​ in the reference mask. The minimum distance is selected to determine how close each predicted point is to its nearest ground truth neighbor. Similarly, for each ground truth point gj​, the nearest predicted point pi​ is identified, and the corresponding minimum distance is calculated. This bidirectional process ensures that both false positives and false negatives are penalized.

For each predicted point $\:{p}_{i}\epsilon\:$P:

- Compute distances to all ground truth points $\:{g}_{j}\epsilon\:$G.
- Select the closest ground truth point using the min operation.

2 $$\:{d}_{min}({p}_{i},G)={min}_{{g}_{\:j}\epsilon\:\mathrm{G}}\left|\right|{p}_{i}-{g}_{\:j}\left|\right|$$

- Do the same for each ground truth point gj, selecting the nearest predicted point.

3 $$\:{d}_{min}({g}_{j},P)={min}_{{P}_{\:i}\epsilon\:\mathrm{P}}\left|\right|{g}_{j}-{p}_{i}\left|\right|$$

### Backward pass (computing the gradients)

To optimize the model, gradients of the loss function need to be computed. Since the loss is based on the minimum Euclidean distance, the partial derivatives with respect to each predicted point pi​ are required. The Euclidean distance function follows a standard formulation, where the gradient is computed as the difference between the predicted and ground truth points, normalized by their Euclidean distance. This gradient guides the model in updating weights to refine segmentation accuracy.

Since the loss is based on the minimum distance to the closest neighbour, we must compute partial derivatives with respect to each predicted point Pi.The Euclidean distance function is:

4 $$\:\:\mathrm{d}(\mathrm{p},\:\mathrm{g})=\:\left|\right|\:\mathrm{p}\:-\mathrm{g}\:\left|\right|=\sqrt{{\left({p}_{x}-{g}_{x}\right)}^{2\:}+{\left({p}_{y}-{g}_{y}\right)}^{2\:}}$$

Its gradient w.r.t. p is:

5 $$\:\frac{\partial\:\mathrm{d}(\mathrm{p},\mathrm{g})}{\partial\:\mathrm{p}}\:=\:\frac{\mathrm{p}-\mathrm{g}}{\left|\left|\mathrm{p}-\mathrm{g}\right|\right|}$$

By combining BCELoss and SDH Loss, we achieve two complementary benefits:

1. Pixel-wise Classification Accuracy (BCELoss).
  Ensures accurate classification of each pixel.
  Handles class imbalance using weighted BCE.
  Works well with deep learning frameworks.

2. Structural and Shape Awareness (SDHL).
  Helps better delineate tumor boundaries.
  Reduces false positives by penalizing boundary mismatches.
  Enhances small tumor segmentation performance.

### Combining BCE loss and SDH loss

The proposed loss function integrates BCE Loss (Binary Cross Entropy) and Sub-Differentiable HausdorffLoss (SDHL) to enhance segmentation performance:

- BCE Loss ensures accurate pixel-wise classification, effectively handling class imbalance and improving the detection of tumor regions.
- SDHL improves structural and shape awareness, helping the model delineate tumor boundaries more precisely. It reduces false positives by penalizing boundary mismatches and enhances the segmentation of small tumors, which are often challenging for traditional losses.

By combining these two loss functions, the model achieves a balance between classification accuracyandstructural alignment, leading to superior segmentation performance in medical imaging applications.

6 $$\:\mathcal{L}={\lambda\:}_{1}{\mathcal{L}}_{BCE}+{\lambda\:}_{2}{\mathcal{L}}_{MHD}$$

The weights were determined through a series of controlled experiments to achieve an optimal balance between region-based accuracy and boundary precision. Initially, both weights were varied in the range $\:\left[\mathrm{0.1,1.0}\right]$in increments of 0.1, and model performance was evaluated using Dice Score and IoU metrics on the validation set. The best results were obtained when $\:{\lambda\:}_{1}=0.6$ and $\:{\lambda\:}_{2}=0.4$, which ensured that the model preserved accurate boundary details while maintaining overall segmentation consistency. This configuration provided the highest Dice Score (90.16%) and IoU (80.78%) with the proposed SDHL + BCE combination.

7 $$\:H\left(P,G\right)=\:\frac{1}{\left|P\right|}\sum\nolimits_{i}{d}_{min}({p}_{i},G)+\:\:\frac{1}{\left|G\right|}\sum\nolimits_{j}{d}_{min}({g}_{j},P)$$

Where:

8 $$\:{d}_{min}({p}_{i},G)={min}_{{g}_{j}\epsilon\:\mathrm{G}}\left|\right|{p}_{i}-{g}_{j}\left|\right|$$

9 $$\:{d}_{min}({g}_{j},P)={min}_{{P}_{i}\epsilon\:\mathrm{P}}\left|\right|{g}_{j}-p\left|\right|$$

To ensure smooth optimization, its differentiability is analyzed. The function is differentiable only when each predicted point has a unique nearest neighbor in the ground truth and vice versa; otherwise, ties in the minimum distance introduce non-differentiable points. The partial derivatives of the loss function are derived by differentiating the minimum Euclidean distance between corresponding points. Specifically, for a predicted point, the gradient is computed using the difference between the coordinates of the prediction and its closest ground truth point, normalized by the Euclidean distance. A similar process is followed for the ground truth points with respect to their nearest predicted neighbors. The final gradients for x, y, u, and v coordinates help in backpropagation, allowing the model to adjust its predictions to minimize segmentation errors. However, the function remains non-differentiable at points where multiple distances are equal, leading to optimization challenges. By integrating this Hausdorff-based loss with BCEWithLogitsLoss, the model benefits from both pixel-wise accuracy and boundary refinement, reducing false positives and improving small tumor segmentation performance.

### Conditions for differentiability

The Hausdorff distance H(P, G) is partially differentiable with respect to the coordinates of $\:{p}_{i}$ or $\:{g}_{j}$only if:

1. The minimum distance $\:{d}_{min}$ ($\:{p}_{i}$, G) is achieved by a unique point in G for all $\:{p}_{i}\epsilon$ P.
2. The minimum distance$\:{d}_{min}\left({g}_{j},P\right)$ is achieved by a unique point in P for all $\:{g}_{j}\epsilon\:$G.

If the minimum distance is achieved by multiple points (i.e„ there are ties), the Hausdorff distance is non-differentiable at those points.

### Partial derivatives (where differentiable)

Assume the conditions for differentiability are satisfied. Leys compute the partial derivatives of.

$\:{H}_{loss}$(P, G) with respect to the coordinates of $\:{p}_{i}$ and $\:{g}_{j}$.

Notation:

- Let $\:{p}_{i}=\left({x}_{i},{y}_{i}\right)$ be a point in P.
- Let $\:{g}_{j}=\:\left({u}_{i},{v}_{i}\right)$be a point in G.
- Let $\:{g}_{k}$ be the unique nearest point in G to $\:{p}_{i}$, i.e., $\:{d}_{min}$ ($\:{p}_{i}$, G) = || $\:{p}_{i}$ - $\:{g}_{k}$ ||.
- Let $\:{p}_{l}$ be the unique point in P to $\:{g}_{j}$, i.e., $\:{d}_{min}$ ($\:{g}_{j}$, P) = || $\:{g}_{j}$ - $\:{p}_{l}$ ||.

Partial Derivative of $\:{H}_{loss}$(P, G) with Respect to$\:{x}_{i}$ :

The Sub-Differential Hausdorff Distance Loss $\:{H}_{loss}$(P, G) depends on only through the term.

$$\:{d}_{min}$$ ($$\:{p}_{i}$$, G). Thus:

10 $$\:\frac{\partial\:{H}_{loss}(P,G)}{\partial\:{x}_{i}}=\:\frac{1}{\left|P\right|}\frac{\partial\:{d}_{min}\left({p}_{i\:,}G\right)}{\partial\:{x}_{i}}$$

The derivative of $\:{d}_{min}\left({p}_{i\:,}G\right)$ with respect to $\:{x}_{i}$ is:

11 $$\:\frac{\partial\:{d}_{min}({p}_{i},G)}{\partial\:{x}_{i}}\:\:=\:\:\:\:\frac{\partial\:}{\partial\:{x}_{i}}\sqrt{{\left({x}_{i}-{u}_{k}\right)}^{2}+{\left({y}_{i}-{v}_{k}\right)}^{2}}$$

12 $$\:=\frac{{x}_{i}-{u}_{k}}{\sqrt{{\left({x}_{i}-{u}_{k}\right)}^{2}+{\left({y}_{i}-{v}_{k}\right)}^{2}}}$$

Thus:

13 $$\:\frac{\partial\:H(P,G)}{\partial\:{x}_{i}}\:\:=\:\frac{1}{\left|P\right|}\frac{{x}_{i}-{u}_{k}}{\sqrt{{\left({x}_{i}-{u}_{k}\right)}^{2}+{\left({y}_{i}-{v}_{k}\right)}^{2}}}$$

Partial Derivative of H (P, G) with Respect to $\:{y}_{i}$:

Similarly:

14 $$\:\frac{\partial\:H(P,G)}{\partial\:{y}_{i}}\:\:=\:\frac{1}{\left|P\right|}\frac{{y}_{i}-{v}_{k}}{\sqrt{{\left({x}_{i}-{u}_{k}\right)}^{2}+{\left({y}_{i}-{v}_{k}\right)}^{2}}}$$

Partial Derivative of H (P, G) with Respect to $\:{u}_{i}$:

The Hausdorff distance H (P, G) depends on $\:{u}_{i}$ only through the term $\:{d}_{min}\left({g}_{j\:,}P\right)$. Thus:

15 $$\:\frac{\partial\:H\left(P,G\right)}{\partial\:{u}_{j}}=\frac{1}{\left|G\right|}\cdot\:\frac{\partial\:{d}_{\mathrm{min}}\left({g}_{j},P\right)}{\partial\:\:{u}_{j}}$$

The derivative of $\:{d}_{min}\left({g}_{j\:,}P\right)$. with respect to $\:{u}_{j}$ is:

16 $$\:\frac{\partial\:{d}_{\mathrm{min}}\left({g}_{j},P\right)}{\partial\:{u}_{j}}=\frac{\partial\:}{\partial\:{u}_{j}}\sqrt{{\left({u}_{j}-{x}_{l}\right)}^{2}+\:{\left({v}_{j}\:-\:{y}_{l}\right)}^{2}}$$

### Summary of partial derivatives

Where the Hausdorff distance is differentiable, the partial derivatives are:

1. With respect to $\:{p}_{i}=\left({x}_{i},{y}_{i}\right)$ :

17 $$\:\frac{\partial\:\mathrm{H}(\mathrm{P},\mathrm{G})}{\partial\:{\mathrm{x}}_{\mathrm{i}}}\:\:=\:\frac{1}{\left|\mathrm{P}\right|}\frac{{\mathrm{x}}_{\mathrm{i}}-{\mathrm{u}}_{\mathrm{k}}}{\sqrt{{\left({\mathrm{x}}_{\mathrm{i}}-{\mathrm{u}}_{\mathrm{k}}\right)}^{2}+{\left({\mathrm{y}}_{\mathrm{i}}-{\mathrm{v}}_{\mathrm{k}}\right)}^{2}}}$$

2. With respect to $\:{g}_{j}=\:\left({u}_{j},{v}_{j}\right)$ :

18 $$\:\frac{\partial\:\mathrm{H}(\mathrm{P},\mathrm{G})}{\partial\:{\mathrm{u}}_{\mathrm{j}}}\:\:=\:\frac{1}{\left|\mathrm{G}\right|}\frac{{\mathrm{u}}_{\mathrm{j}}-{\mathrm{x}}_{\mathrm{l}}}{\sqrt{{\left({\mathrm{u}}_{\mathrm{j}}-{\mathrm{x}}_{\mathrm{l}}\right)}^{2}+{\left({\mathrm{v}}_{\mathrm{j}}-{\mathrm{y}}_{\mathrm{l}}\right)}^{2}}}$$

19 $$\:\frac{\partial\:\mathrm{H}(\mathrm{P},\mathrm{G})}{\partial\:{\mathrm{v}}_{\mathrm{j}}}\:\:=\:\frac{1}{\left|\mathrm{G}\right|}\frac{{\mathrm{v}}_{\mathrm{j}}-{\mathrm{y}}_{\mathrm{l}}}{\sqrt{{\left({\mathrm{u}}_{\mathrm{j}}-{\mathrm{x}}_{\mathrm{l}}\right)}^{2}+{\left({\mathrm{v}}_{\mathrm{j}}-{\mathrm{y}}_{\mathrm{l}}\right)}^{2}}}$$

### Non-differentiability at ties

If the minimum distance $\:{d}_{min}\left({p}_{i\:,}G\right)$ or $\:{d}_{min}\left({g}_{j\:,}P\right)$. is achieved by multiple points, the Hausdorff distance is non-differentiable at those points. This is because the minimum function introduces “kinks” or “corners” in the distance function.

### Compute the gradient of H (P, G)

The gradient of H (P, G) with respect to the model parameters $\:{\uptheta\:}$ is:

20 $$\:\mathrm{H}\left(\mathrm{P},\mathrm{G}\right)=\:\frac{1}{\left|\mathrm{P}\right|}\sum\nolimits_{\mathrm{i}}{{\nabla\:}_{{\uptheta\:}}\mathrm{d}}_{\mathrm{m}\mathrm{i}\mathrm{n}}({\mathrm{p}}_{\mathrm{i}},\mathrm{G})+\:\:\frac{1}{\left|\mathrm{G}\right|}\sum\nolimits_{\mathrm{j}}{{\nabla\:}_{{\uptheta\:}}\mathrm{d}}_{\mathrm{m}\mathrm{i}\mathrm{n}}({\mathrm{g}}_{\mathrm{j}},\mathrm{P})$$

Compute$$\:{{\nabla\:}_{{\uptheta\:}}\mathrm{d}}_{\mathrm{m}\mathrm{i}\mathrm{n}}\left({\mathrm{p}}_{\mathrm{i}},\mathrm{G}\right):$$.

For each predicted point$\:{\mathrm{p}}_{\mathrm{i}}$, let $\:{\mathrm{g}}_{\mathrm{k}}$be the nearest ground truth point. Then:

21 $$\:{{\nabla\:}_{{\uptheta\:}}\mathrm{d}}_{\mathrm{m}\mathrm{i}\mathrm{n}}\left({\mathrm{p}}_{\mathrm{i}},\mathrm{G}\right)\:=\:\frac{{\mathrm{p}}_{\mathrm{i}}\:-\:\:{\mathrm{g}}_{\mathrm{k}}}{\left|\right|\:{\mathrm{p}}_{\mathrm{i}}\:-\:\:{\mathrm{g}}_{\mathrm{k}}\:\left|\right|}{\nabla\:}_{{\uptheta\:}}{\mathrm{p}}_{\mathrm{i}}$$

Compute$$\:{{\nabla\:}_{{\uptheta\:}}\mathrm{d}}_{\mathrm{m}\mathrm{i}\mathrm{n}}({\mathrm{g}}_{\mathrm{j}},\mathrm{P}$$) :

For each predicted point$\:{\mathrm{g}}_{\mathrm{j}}$, let $\:{\mathrm{p}}_{\mathrm{i}}$be the nearest predicted point. Then:

22 $$\:{{\nabla\:}_{{\uptheta\:}}\mathrm{d}}_{\mathrm{m}\mathrm{i}\mathrm{n}}\left({\mathrm{g}}_{\mathrm{j}},\mathrm{P}\right)=\frac{{\mathrm{g}}_{\mathrm{j}}\:-\:\:{\mathrm{p}}_{\mathrm{l}}}{\left|\right|\:{\mathrm{g}}_{\mathrm{j}}\:-\:\:{\mathrm{p}}_{\mathrm{l}}\:\left|\right|}{\nabla\:}_{{\uptheta\:}}{\mathrm{p}}_{\mathrm{l}}$$

Adam (Adaptive Moment Estimation) is an optimization algorithm that combines the benefits of momentum and adaptive learning rates. It maintains two moving averages of the gradient:

1. First moment (mean): $\:{\mathrm{m}}_{\mathrm{t}}$
2. Second moment (uncentered variance): $\:{\mathrm{v}}_{\mathrm{t}}$

The update steps for Adam are **Step1: Initialize**:

$\:{\mathrm{m}}_{0}\:=$ 0 (first moment vector).

$$\:{\mathrm{v}}_{0\:\:\:\:}=\:$$0 (second moment).

$$\:\:\mathrm{t}\:\:\:\:=0\:\:\left(\mathrm{t}\mathrm{i}\mathrm{m}\mathrm{e}\:\mathrm{s}\mathrm{t}\mathrm{e}\mathrm{p}\right)$$

#### Step 2:

At each iteration of t.

1. Compute the gradient $\:{\mathrm{g}}_{\mathrm{t}}$ = $\:{\nabla\:}_{{\uptheta\:}}$H (P, G).
2. Update the first moment:

23 $$\:{\mathrm{m}}_{\mathrm{t}}={{\upbeta\:}}_{1}{\mathrm{m}}_{\mathrm{t}-1}+\left(1-{{\upbeta\:}}_{1}\right){\mathrm{g}}_{\mathrm{t}}$$

3. Update the second moment:

24 $$\:{\mathrm{v}}_{\mathrm{t}}={{\upbeta\:}}_{2}{\mathrm{v}}_{\mathrm{t}-1}+\left(1-{{\upbeta\:}}_{2}\right){\mathrm{g}}_{\mathrm{t}}^{2}$$

4. Bias-correct the first moment:

25 $$\:\widehat{{\mathrm{m}}_{\mathrm{t}}}=\frac{{\mathrm{m}}_{\mathrm{t}}}{1-{{\upbeta\:}}_{1}^{\mathrm{t}}}$$

5. Bias-correct the second moment:

26 $$\:\widehat{{\mathrm{v}}_{\mathrm{t}}}=\frac{{\mathrm{v}}_{\mathrm{t}}}{1-{{\upbeta\:}}_{2}^{\mathrm{t}}}$$

6. Update the parameters:

27 $$\:{{\uptheta}}_{\mathrm{t}}={{\uptheta}}_{\mathrm{t}-1}-{\upeta\:}\cdot\:\frac{\widehat{{\mathrm{m}}_{\mathrm{t}}}}{\sqrt{\widehat{{\mathrm{v}}_{\mathrm{t}}}}+{\epsilon}}$$

Where:

- $\:{{\upbeta\:}}_{1}$and $\:{{\upbeta\:}}_{2}$ are exponential decay rates (typically $\:{{\upbeta\:}}_{1}$= 0.9, $\:{{\upbeta\:}}_{2}=$ 0.999).
- $\:{\upeta\:}\:$is the learning rate.
- $\:{\epsilon}\:$is a small constant for numerical stability (e.g., $\:{\epsilon}$ =$\:{10}^{-8}$).

#### Step 3:

Training Algorithm.

The training process can be summarized as follows:

1. Input: Training dataset {($\:{\mathrm{X}}_{1}$, $\:{\mathrm{Y}}_{1}$), {($\:{\mathrm{X}}_{2}$, $\:{\mathrm{Y}}_{2}$),…….,{($\:{\mathrm{X}}_{\mathrm{N}}$, $\:{\mathrm{Y}}_{\mathrm{N}}$), }, where N is the numberof training samples.
2. Initialize: Network parameters$\:{\uptheta\:}$, Adam moments$\:{\mathrm{m}}_{0}=0\:$,$\:{\mathrm{v}}_{0}=0$, and time step t = 0.
3. Repeat for each epoch:

- Shuffle the training data.
- For each mini-batch {{($\:{\mathrm{X}}_{\mathrm{b}}$, $\:{\mathrm{Y}}_{\mathrm{b}}$)}:
- Compute the predicted segmentation $\:\widehat{{\mathrm{Y}}_{\mathrm{b}}}={\mathrm{f}}_{0\:}\left({\mathrm{X}}_{\mathrm{b}}\right)\:$.
- Extract the predicted boundary points P and ground truth boundary points from $\:\widehat{{\mathrm{Y}}_{\mathrm{b}}}$and$\:{\mathrm{Y}}_{\mathrm{b}}$, respectively.
- Compute the Hausdorffdistanceloss H(P, G).
- Compute the gradient $\:{\mathrm{g}}_{\mathrm{t}}$ = $\:{\nabla\:}_{{\uptheta\:}}$ H (P, G).
- Update the Adam moment:

28 $$\:{\mathrm{m}}_{\mathrm{t}}={{\upbeta\:}}_{1}{\mathrm{m}}_{\mathrm{t}-1}+\left(1-{{\upbeta\:}}_{1}\right){\mathrm{g}}_{\mathrm{t}}$$

29 $$\:{\mathrm{v}}_{\mathrm{t}}={{\upbeta\:}}_{2}{\mathrm{v}}_{\mathrm{t}-1}+\left(1-{{\upbeta\:}}_{2}\right){\mathrm{g}}_{\mathrm{t}}^{2}$$

- Bias-correct the first moment:

30 $$\:\widehat{{\mathrm{m}}_{\mathrm{t}}}=\frac{{\mathrm{m}}_{\mathrm{t}}}{1-{{\upbeta\:}}_{1}^{\mathrm{t}}}$$

31 $$\:\widehat{{\mathrm{v}}_{\mathrm{t}}}=\frac{{\mathrm{v}}_{\mathrm{t}}}{1-{{\upbeta\:}}_{2}^{\mathrm{t}}}$$

- Update the parameters:

32 $$\:{{\uptheta\:}}_{\mathrm{t}}={{\uptheta\:}}_{\mathrm{t}-1}-{\upeta\:}\cdot\:\frac{\widehat{{\mathrm{m}}_{\mathrm{t}}}}{\sqrt{\widehat{{\mathrm{v}}_{\mathrm{t}}}}+{\epsilon}}$$

- Increment the time step: t = t + 1.

Output: Trained network parameters$\:{\uptheta\:}$.

## Proposed model with novel loss function

**Fig. 4:**
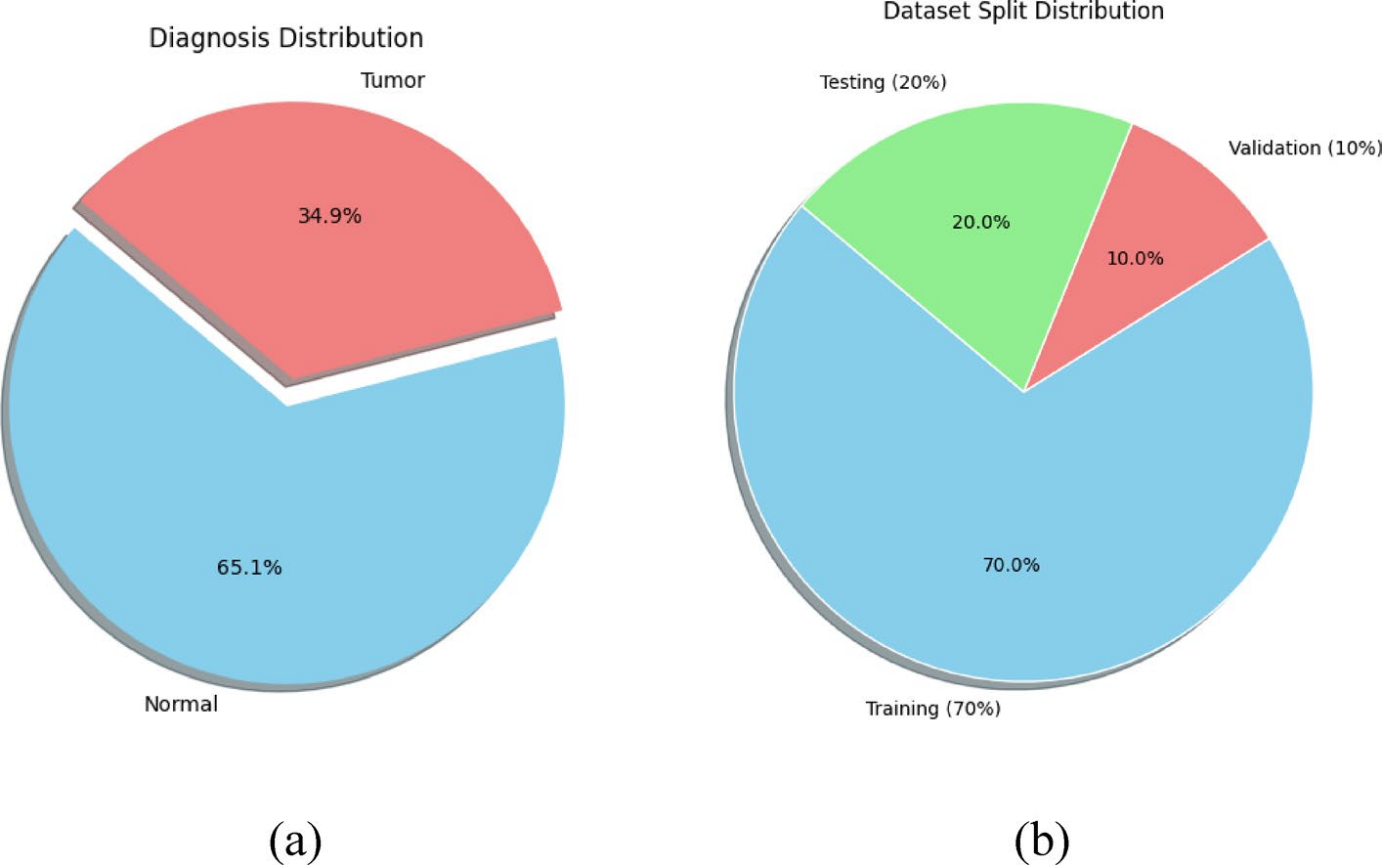
**(a)** Dataset distribution **(b)** Train-Test split.

The model is trained using the LGG Brain Tumor Segmentation Dataset **(**https://www.kaggle.com/datasets/mateuszbuda/lgg-mri-segmentation), which contains MRI scans categorized as tumor and normal. As shown in Fig. 4, the dataset exhibits a class imbalance (34.9% tumor vs. 65.1% normal). To mitigate this, we performed data augmentation (rotation, flipping, and intensity normalization), ensuring balanced representation and enhanced generalization.

**Fig. 5:**
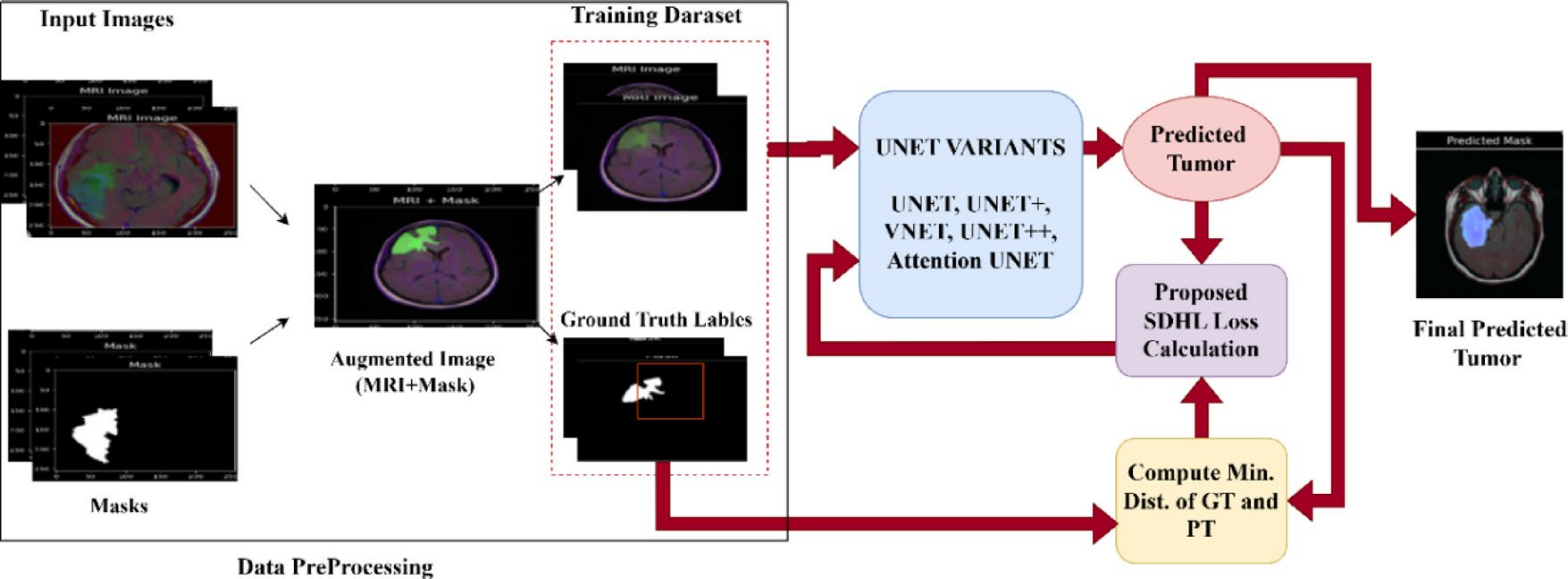
Working of proposed loss function on UNET variants.

1. Training-Validation Split: The dataset is divided into 70% training, 20% testing, and 10% validation subsets, ensuring both robust training and unbiased evaluation.
2. Training Configuration: The model training uses the Adam optimizer with an initial learning rate of 0.001, batch size of 32, and 200 epochs. The ReLU activation introduces non-linearity and avoids vanishing gradients, while a dropout rate of 0.1 minimizes overfitting.

3. Loss Function and Optimization: The proposed Sub-Differentiable Hausdorff Loss (SDHL) is integrated with Binary Cross Entropy (BCE) loss to improve segmentation boundary precision. The training workflow is clearly depicted in Algorithm 1 and Fig. 3, which include:
  Finding the closest point between prediction (P) and ground truth (G).
  Computing the final Hausdorff Distance.
  Forward pass (loss computation).
  Backward pass (gradient computation).
  Combining BCE and SDHL for total loss minimization.

4. Differentiability and Gradient Flow: Additional mathematical explanations have been added to clarify conditions for differentiability, partial derivatives, and gradient computation, which are essential for ensuring stable backpropagation.
5. Model Integration: The proposed loss function is implemented and tested on U-Net and its variants, as illustrated in Fig. 5, demonstrating improved convergence behavior and segmentation accuracy.

These detailed clarifications have been incorporated in the revised manuscript to ensure that the training workflow and optimization process are explicitly understandable and reproducible.

The working of proposed loss function on UNET variants is shown in Fig. 5. The process begins with data preprocessing, where MRI images and corresponding masks undergo augmentation before being divided into training and test sets. Various segmentation models, including UNET, UNET+, VNET, UNET++, and Attention UNET, are employed to process the input images and generate tumor predictions. A novel loss function, termed SDHL Loss, is introduced, which enhances segmentation accuracy by computing the minimum distance between the Ground Truth (GT) and Predicted Tumor (PT), ensuring better spatial consistency. Once the model generates the final predicted masks, performance is evaluated using key metrics such as Accuracy, Precision, Recall, F1 Score, IoU, Dice Score, and Matthews Correlation Coefficient. The SDHL Loss function is designed to refine segmentation by focusing on boundary alignment and reducing misclassification errors through distance-based constraints.

### Data pre-processing and augmentation

Data pre-processing and augmentation techniques for training and inference in a brain tumor segmentation task using the Albumentationslibrary. During training, various transformations are applied to enhance dataset diversity and improve model generalization. The training pipeline includes resizing images to a predefined height and width, followed by random brightness and contrast adjustments to handle variations in lighting conditions. Additionally, horizontal and vertical flips are introduced with a 50% probability to increase variability in the dataset. Finally, the images are converted into PyTorch tensors for model compatibility. In contrast, the inference pipeline applies only resizingandtensor conversion, ensuring that test images retain their original characteristics for accurate predictions.

### Proposed loss function

The proposed SDHL Loss (Spatial Distance-based Hausdorff Loss) improves segmentation by minimizing the worst-case deviation between predicted tumor masks and ground truth labels. It works by computing the Hausdorff Distance, which measures the maximum discrepancy between two point sets. The process begins by finding the closest point for each ground truth pixel in the predicted mask and determining the minimum distance. The maximum of these minimum distances is recorded, representing how far any ground truth point deviates from the prediction. This process is repeated in reverse, where each predicted pixel is mapped to its nearest ground truth point, and the maximum minimum distance is computed. The final loss is determined by averaging these distances, ensuring that both the prediction and ground truth are spatially aligned. Unlike traditional losses such as Dice or Cross-Entropy, SDHL Loss explicitly penalizes boundary misalignment, improving segmentation accuracy by focusing on spatial consistency and reducing false positives and negatives.

After defining the structure and working mechanism of the proposed model, the subsequent section describes the experimental setup, dataset, and evaluation metrics used to validate the effectiveness of the proposed SDHL-based framework.

## Experimental outcomes

### Dataset

The LGG Segmentation Dataset **(**https://www.kaggle.com/datasets/mateuszbuda/lgg-mri-segmentation) is a widely used medical imaging dataset for brain tumor segmentation and classification. In Fig. 4. the first pie chart illustrates the proportion of tumor and normal cases, showing that 34.9% of the data corresponds to tumor cases, while 65.1% represents normal cases, highlighting a class imbalance where normal brain scans dominate. This imbalance can affect model training by making it biased toward the majority class. The second pie chart depicts the dataset split, where 70% of the data is allocated for training, 20% for testing, and 10% for validation. This division ensures the model is trained on a substantial portion of data while also being evaluated on separate validation and test sets to assess its generalization ability. Understanding these distributions is crucial for developing an effective and unbiased machine-learning model for brain tumor segmentation.

### Experimental setup

The experimental setup for the brain tumor segmentation model is designed to optimize performance using the Adam optimizer, which efficiently combines momentum and adaptive learning rates. The ReLU activation function is employed to introduce non-linearity and prevent vanishing gradient issues. The model is trained with a learning rate of 0.001, ensuring a balance between convergence speed and stability. To mitigate overfitting, a dropout rate of 0.1 is applied during training. The model undergoes training for 200 epochs, allowing it to learn complex patterns in the data. A batch size of 32 is used to ensure efficient training while leveraging the computational capabilities of the hardware. This setup ensures effective parameter updates, stability, and generalization for accurate tumor segmentation.

### Evaluation metrics

1. Accuracy: Itmeasures the proportion of correctly classified instances among all instances.

33 $$\:\mathrm{Accuracy}=\frac{\mathrm{TP}+\mathrm{TN}}{\mathrm{TP}+\mathrm{TN}+\mathrm{FP}+\mathrm{FN}}$$

2. Precision: It measures how many of the predicted positive cases are actually positive.

34 $$\:\mathrm{Precision}=\frac{\mathrm{TP}}{\mathrm{TP}+\mathrm{FP}}$$

3. Recall: It measures how many actual positive cases were correctly identified.

35 $$\:\mathrm{Recall}=\frac{\mathrm{TP}}{\mathrm{TP}+\mathrm{FN}}$$

4. Dice Similarity Coefficient: It is a measure of overlap between the predicted and ground truth segmentations.

36 $$\:Dice\:Score=\frac{2\times\:\left|{Y}_{target}\cap\:{Y}_{Pred}\right|}{\lceil{Y}_{target}\rceil+\lceil{Y}_{Pred}\rceil}$$

5. F1-Score: Itis the harmonic mean of precision and recall

37 $$\:\mathrm{F1\:Score}=\frac{2\times\:\mathrm{Precision}\times\:\mathrm{Recall}}{\mathrm{Precision}+\mathrm{Recall}}$$

6. Intersection over Union: measures the overlap between predicted and ground truth segmentations.

38 $$\:\mathrm{IoU}=\frac{\left|\mathrm{Ytarget}\cap\:\mathrm{Ypred}\right|}{\left|\mathrm{Ytarget}\cup\:\mathrm{Ypred}\right|}$$

7. Matthews Correlation Coefficient: It is a balanced measure that considers all four confusion matrix components.

39 $$\:\mathrm{Matthews\:Correlation\:Coefficient}=\frac{\left(\mathrm{TP}\times\:\mathrm{TN}-\mathrm{FP}\times\:\mathrm{FN}\right)}{\sqrt{\left(\mathrm{TP}+\mathrm{FP}\right)\left(\mathrm{TP}+\mathrm{FN}\right)\left(\mathrm{TN}+\mathrm{FP}\right)\left(\mathrm{TN}+\mathrm{FN}\right)}}$$

### Results and discussion

**Fig. 6:**
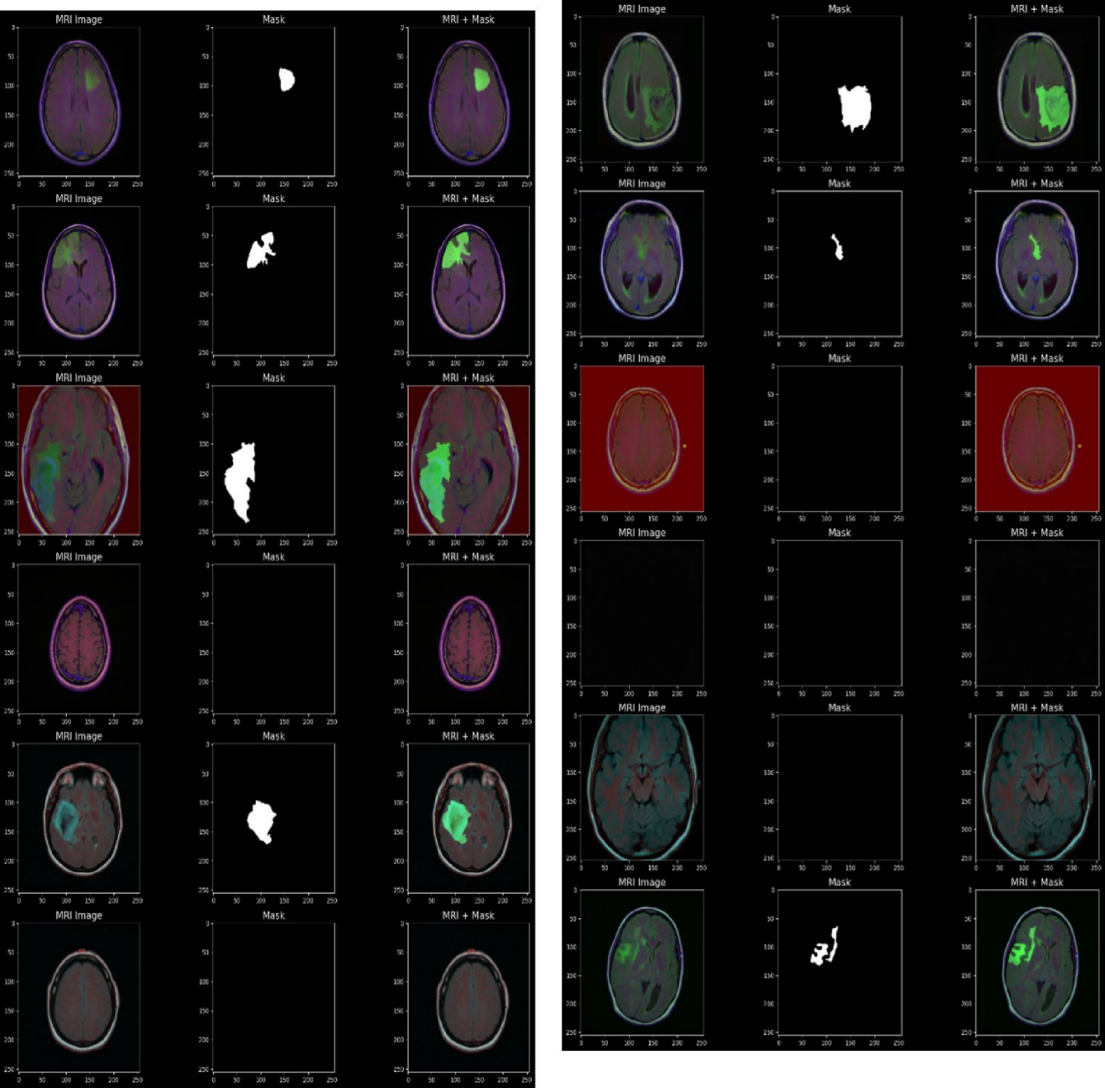
Input images.

The training process begins with a dataset containing pairs of input images and their corresponding ground truth labels is shown in Fig. 6. Initially, the network parameters, along with the Adam optimization moments and time step, are set to their default values. During each training epoch, the dataset is shuffled to ensure variability in learning. The model processes the data in small mini-batches, where each input batch is passed through the network to generate predicted segmentations. From these segmentations, the boundary points of the predicted and ground truth masks are extracted.

**Fig. 7:**
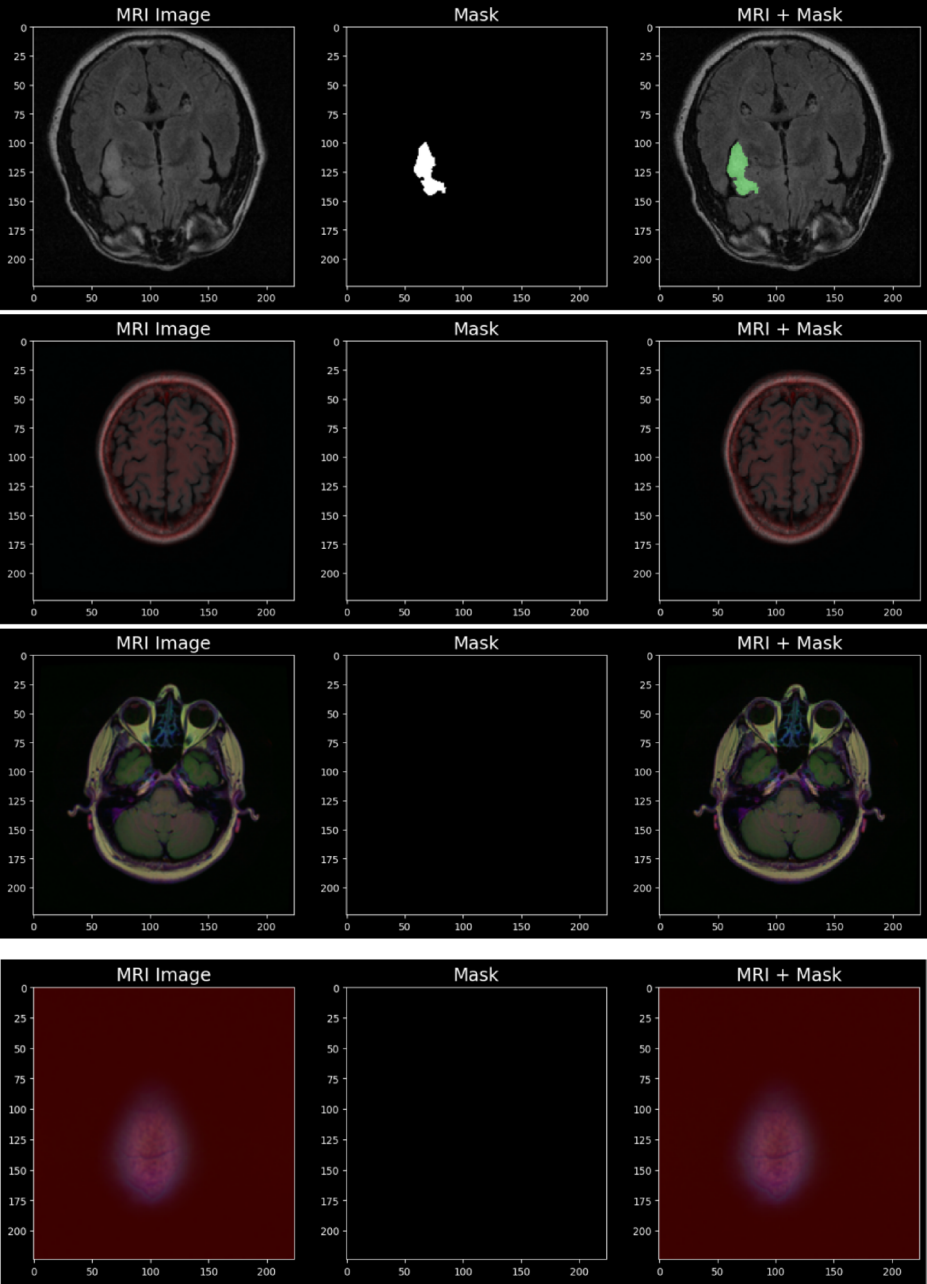
Augmented images.

The MRI scans from the LGG (Low-Grade Glioma) dataset for brain tumor segmentation, structured into three columns is shown in Fig. 7. The first column displays raw MRI images capturing brain structures, the second column shows ground truth segmentation masks where white regions indicate tumor areas, and the third column overlays the mask onto the MRI scan, highlighting tumors in green. Some images contain clear tumor regions, while others show no abnormalities. The variation in mask shapes and sizes reflects the diverse tumor characteristics.

**Table 2:** Semantic segmentation metrics of UNET variant models with new loss function.

Model	Loss function	Evaluation Metrics				
		Accuracy	Precision	Recall	Dice_score	IoU
U-Net	SDHL + BCE	99.49	87.58	87.65	85.77	77.54
U-Net+	SDHL + BCE	99.52	87.96	88.03	86.17	77.86
V Net	SDHL + BCE	99.58	88.14	89.68	87.98	78.97
U-Net++	SDHL + BCE	99.67	89.98	90.06	88.59	79.57
Attention U Net	SDHL + BCE	99.71	91.06	91.85	90.16	80.78

The Table 2 presents a comparative analysis of segmentation models—U-Net, U-Net+, V-Net, U-Net++, and Attention U-Net, trained using a combined loss function of SDHL (Spatial Directional Hybrid Loss) and BCE (Binary Cross Entropy). These models were evaluated on how accurately they segment tumor areas in MRI images at the pixel level, using key performance metrics: Accuracy, Precision, Recall, Dice Score, and Intersection over Union (IoU). Accuracy indicates the percentage of correctly classified pixels (both tumor and non-tumor), Precision shows how many of the predicted tumor pixels are actually tumor, Recall (or Sensitivity) reflects how many actual tumor pixels were correctly identified, and the F1 Score provides a balance between Precision and Recall. Dice Score and IoU measure the overlap between the predicted and actual tumor regions, with Dice focusing on pixel agreement and IoU on the region intersection. The results demonstrate a consistent improvement in performance as the model architectures evolve. U-Net begins with an accuracy of 99.49%, precision of 87.58%, and Dice Score of 85.77%, while the Attention U-Net outperforms all others with the highest accuracy of 99.71%, precision of 91.06%, recall of 91.85%, Dice Score of 90.16%, and IoU of 80.78%. The integration of attention mechanisms in Attention U-Net significantly enhances its ability to capture contextual and boundary information, making it the most effective model for accurate tumor segmentation in MRI images. Finally, the goal is to assess how well each model can segment (mark) the tumor region in MRI scans, and the results clearly show that more advanced models paired with a robust loss function offer better segmentation performance.

**Fig. 8:**
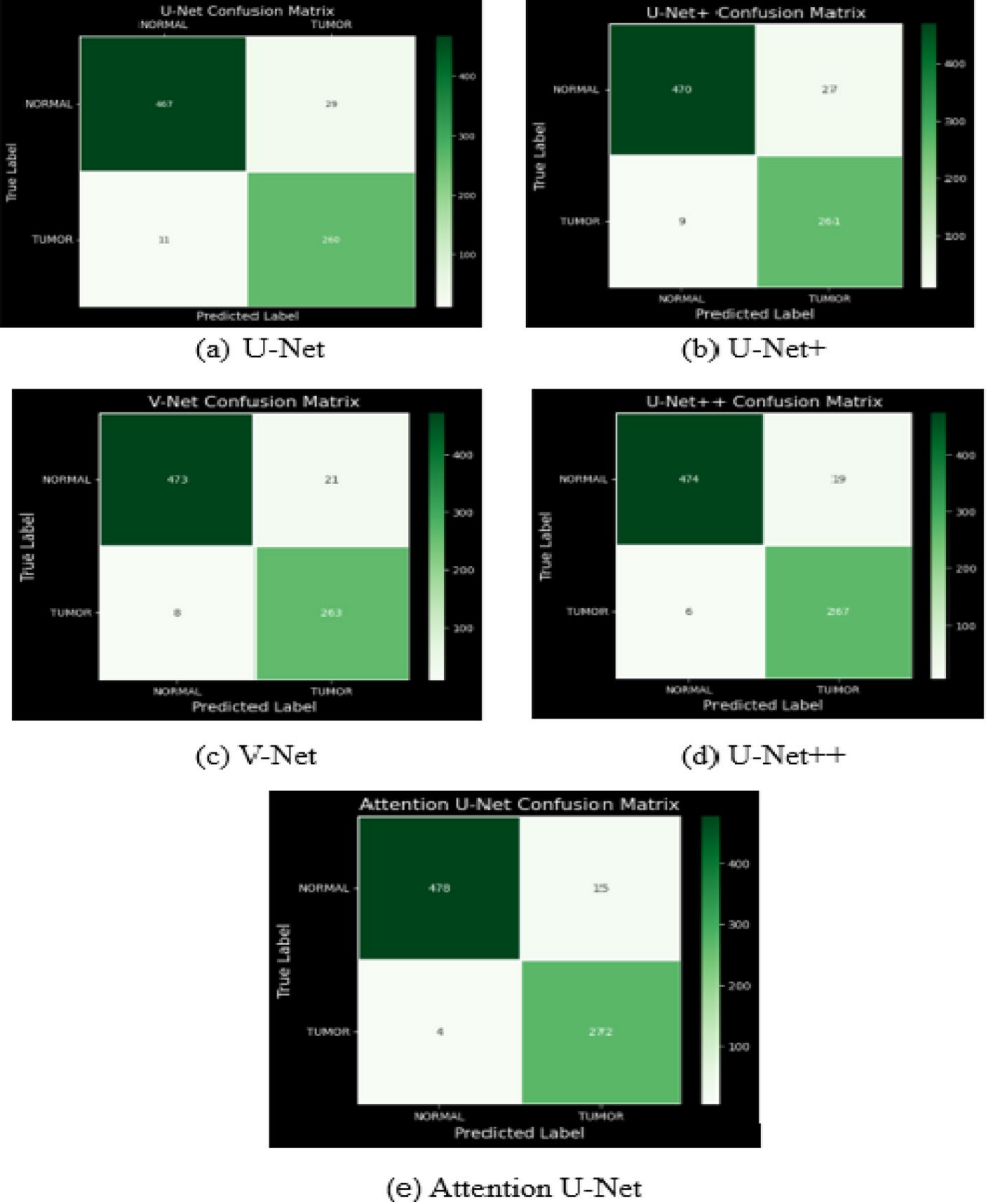
Confusion matrix of various models with new loss function.

The confusion matrices for different deep learning models used for brain tumor segmentation, including U-Net, U-Net+, V-Net, U-Net++, and Attention U-Net is shown in Fig. 8. Each confusion matrix visually represents the performance of a model in classifying MRI images into “Normal” and “Tumor” categories. The diagonal elements indicate correct classifications, while off-diagonal elements represent misclassifications. Darker shades in the confusion matrices correspond to higher values, indicating better model performance. Among the models, Attention U-Net appears to achieve the best results, with fewer misclassified cases and higher accuracy in distinguishing tumor and normal regions. By comparing these matrices, one can assess the effectiveness of each model in brain tumor segmentation.

**Table 3:** Different loss metrics comparison on various UNET variants.

Method	Loss	IoU	Dice
**UNet ** ^23^	EXP	71.5	79.01
	BCE	69.6	76.91
	Dice	70.6	78.01
	Symmetric focal	68.8	76.02
	Asymmetric focal	67.64	74.74
	**Proposed loss function with BCE loss**	**77.54**	**85.77**
**UNet++ ** ^23^	EXP	77.26	85.37
	BCE	76	83.98
	Dice	75.28	83.18
	Symmetric focal	70.86	78.3
	Asymmetric focal	72.21	79.79
	**Proposed loss functionwith BCE loss**	**79.57**	**88.59**
**AttUNet ** ^23^	EXP	70.44	75.6
	BCE	68.42	77.46
	Dice	70.1	75.44
	Symmetric focal	68.27	74.32
	Asymmetric focal	67.26	79.96
	**Proposed loss functionwith BCE loss**	**80.78**	**90.16**

The Table 3 compares the performance of three segmentation models: UNet, UNet++, and AttUNet using various loss functions, with performance measured by Intersection over Union (IoU) and Dice Score. Among standard loss functions, EXP (Exponential Loss)generally performs well, while BCE (Binary Cross-Entropy), Dice Loss, andFocal Loss variantsshow varied effectiveness. The Proposed Loss Function consistently improves IoU, achieving 85.77% in UNet, 88.59% in UNet++, and 90.16% in AttUNet, significantly outperforming other losses. While the Dice Score improvements are less dramatic, AttUNet benefits the most, reaching 80.78%, the highest among all configurations. Traditional losses like EXP and BCE are effective, but focal losses show inconsistent results, with Asymmetric Focal Loss providing a relatively high Dice Score (79.96%) in AttUNet.

**Fig. 9:**
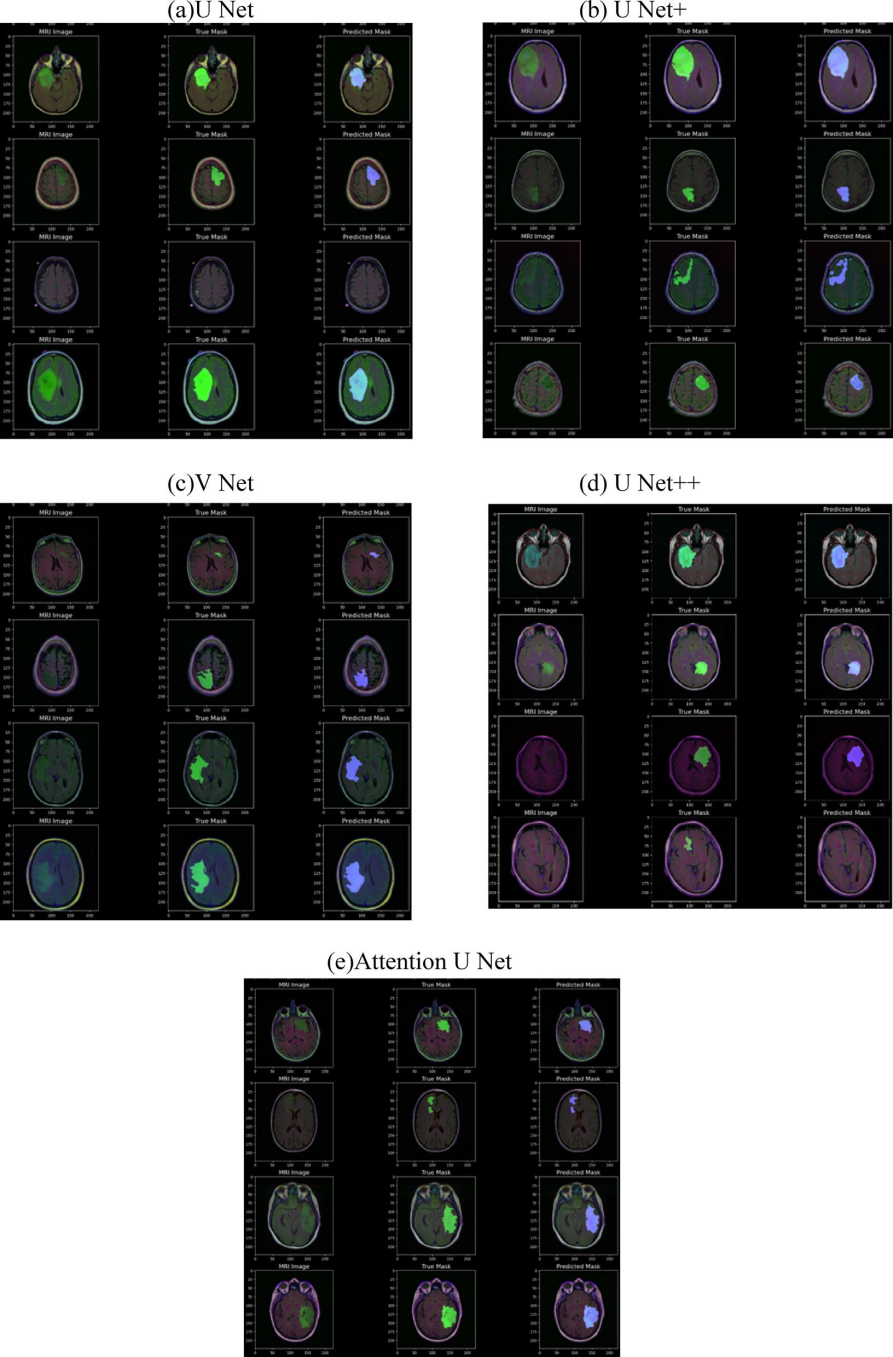
Test diagnosed images.

**Fig. 10:**
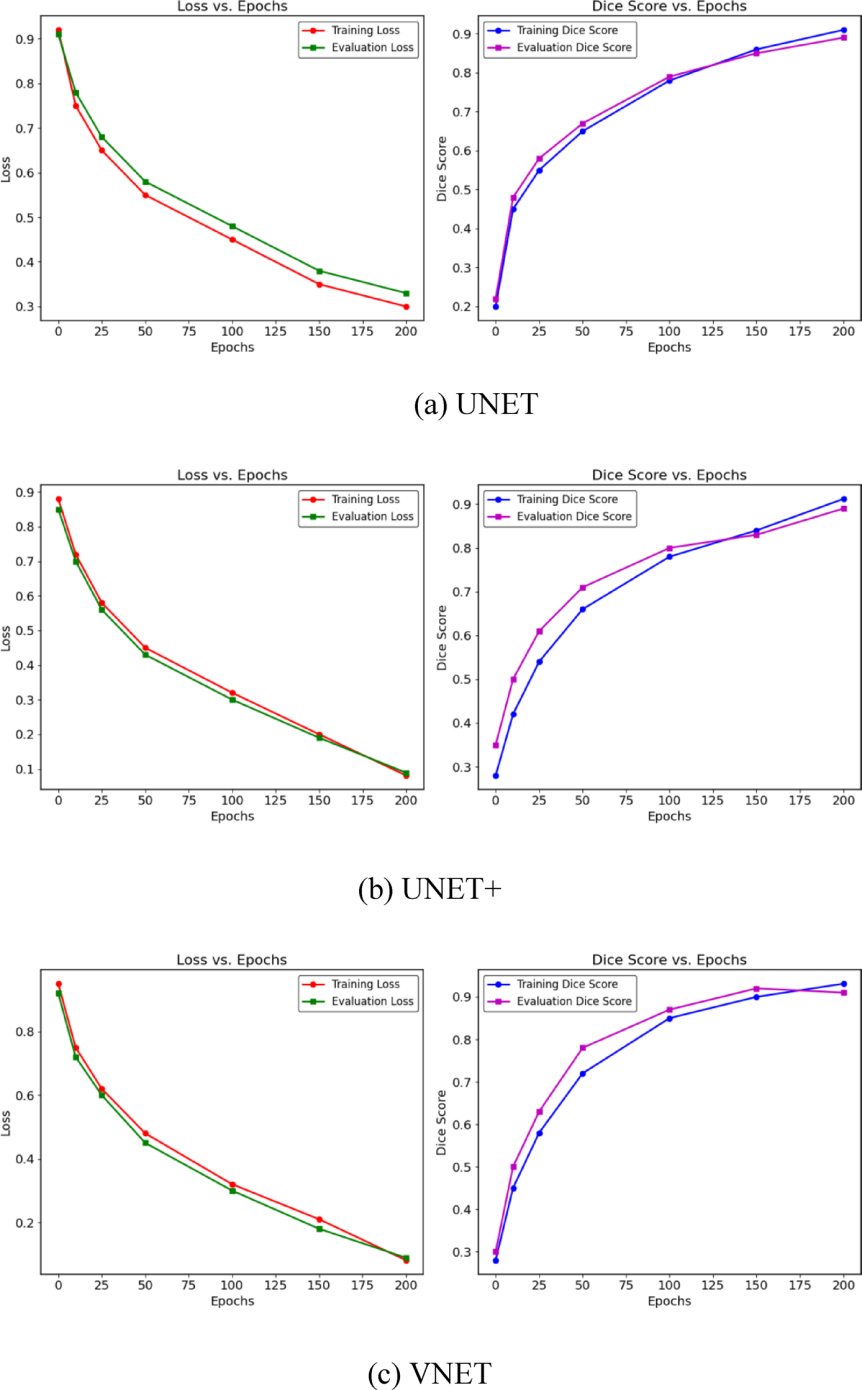

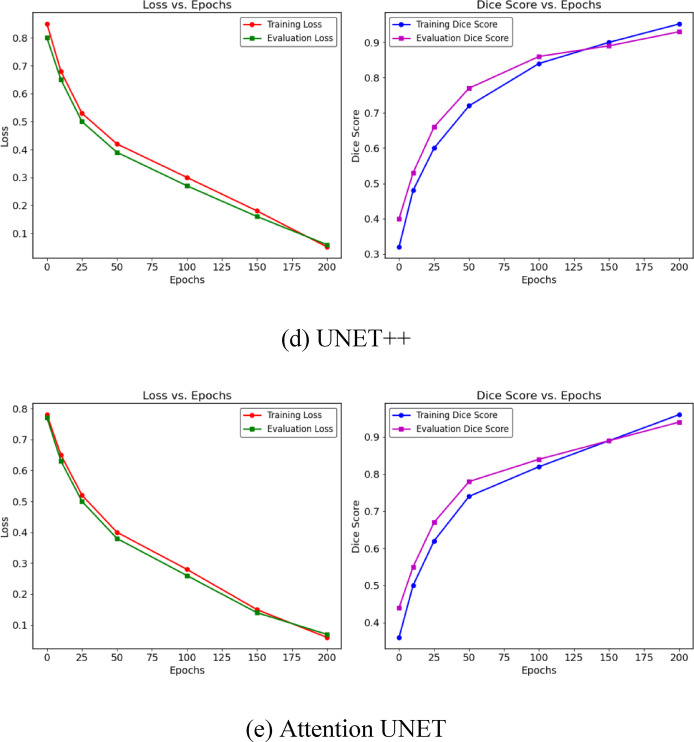
Loss and dice score plots for UNET, UNET+, VNET, UNET++, attention UNET with (SDHL + BCE) loss function.

The results of a brain tumor segmentation test images using (a) U Net, (b) U Net+, (c) V Net, (d) U Net + + and (e) Attention U Net is shown in Fig. 9. It consists of three columns: the first column displays the original MRI images, the second column shows the true tumor masks (ground truth annotations), and the third column contains the predicted tumor masks generated by a segmentation model. Each row represents a different test case. The true masks highlight the actual tumor regions as annotated by experts, while the predicted masks indicate the model’s segmentation output. By comparing the true and predicted masks, one can assess the model’s accuracy in identifying tumor regions. Variations in the overlap between the predicted and true masks suggest the model’s performance, with closer alignment indicating better segmentation accuracy.The loss and Dice Score plots for UNET, UNET+, VNET, UNET++, Attention UNET with (SDHL + BCE) Loss function are shown in Fig. 10.

**Fig. 11:**
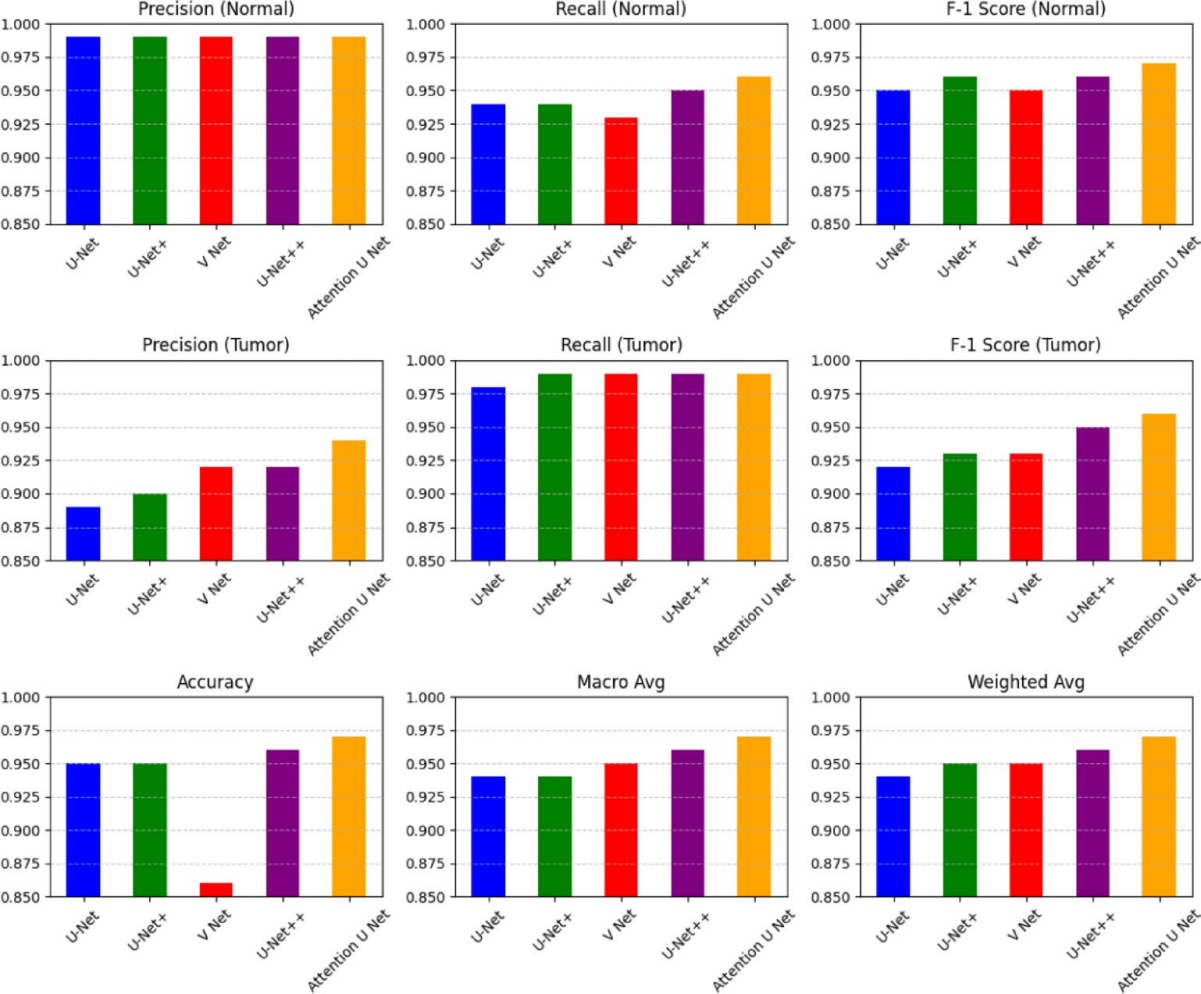
Diagnosis classification metrics of the various models with new loss function.

**Fig. 12:**
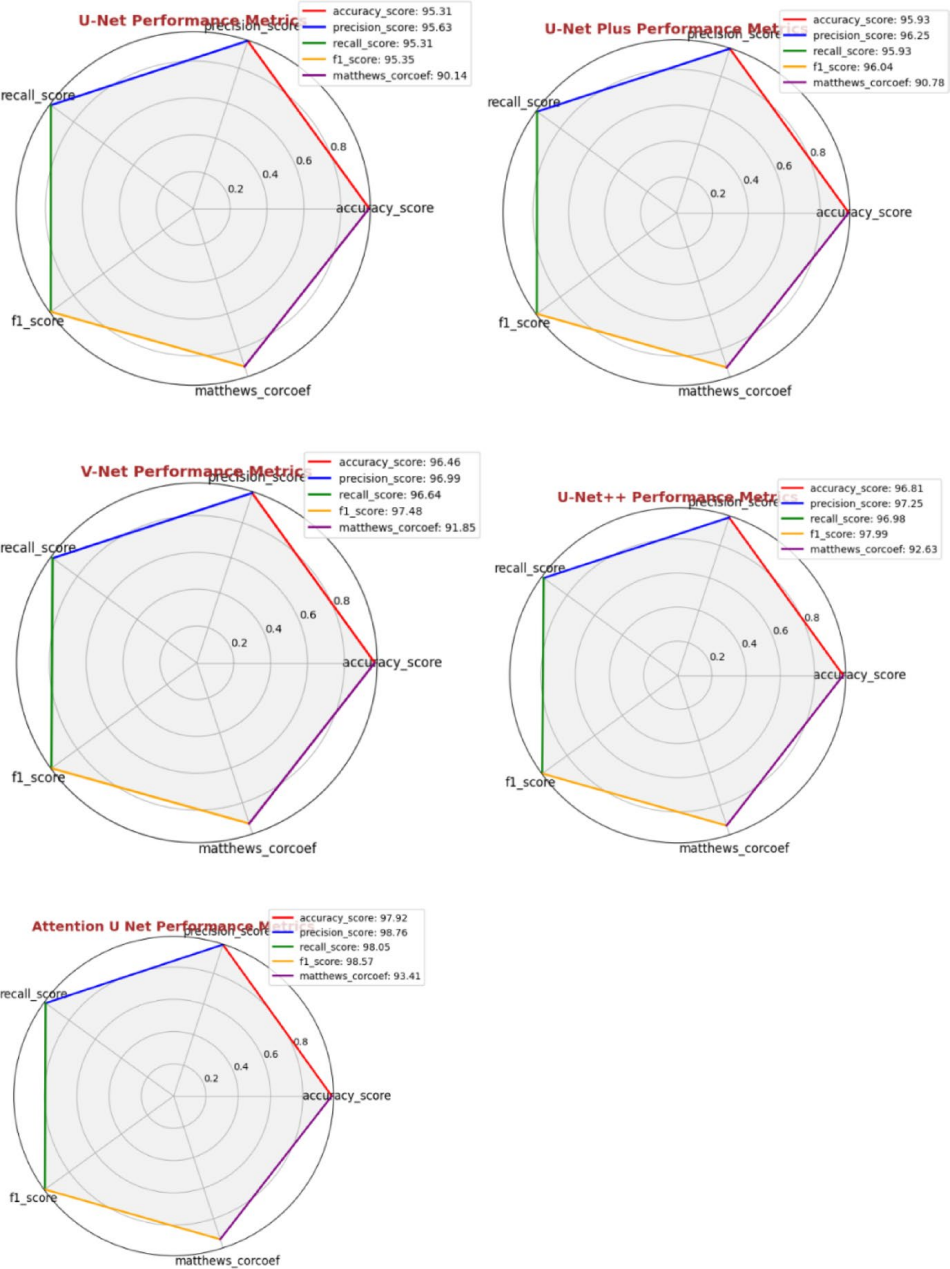
Performance metrics of UNET variants.

The multiple bar charts comparing the performance of different models is shown in Fig. 11. Each chart represents a distinct evaluation metric, offering insight into the models’ effectiveness. These metrics include Precision (Normal), which measures the accuracy of positive predictions for the “Normal” class, and Recall (Normal), which assesses the model’s ability to correctly identify all “Normal” instances. The F1 Score (Normal) balances these two aspects.Similarly, the diagram evaluates Precision (Tumor), measuring the accuracy of positive predictions for the “Tumor” class, and Recall (Tumor), reflecting the model’s capability to correctly identify all “Tumor” instances. The F1 Score (Tumor) provides a balanced measure of precision and recall for this class. Additionally, overall model performance is assessed through Accuracy, which measures the overall correctness, Macro Average, which averages performance across all classes, and Weighted Average, which combines precision, recall, and F1-score based on class distribution.The comparison includes multiple models or architectures, such as U-Net, U-Net++, V-Net, and Attention U-Net. The bars in the charts represent the performance scores, where higher values indicate better performance. Based on the diagram, the Attention U-Net appears to perform consistently well across all metrics, often surpassing or equaling the other models. However, variations in performance are noted, particularly in Recall (Normal) and Precision (Tumor). Performance metrics of UNET variant models is shown in Fig. 12.

The training and validation performance of various deep learning models U-Net, U-Net+, V-Net, U-Net++, and Attention U-Net used for segmenting tumor regions in MRI images is shown in Fig. 12. These plots visualize key metrics including Accuracy, Precision, Recall, F1 Score, and Matthews Correlation Coefficient (MCC), which are essential for tracking how effectively each model learns to segment tumors over time. U-Net serves as a strong baseline with balanced performance, while U-Net + shows slight improvements in generalization. V-Net demonstrates a notable boost in all metrics, indicating more robust learning. U-Net + + further refines this with improved feature representation, leading to better segmentation predictions. Among all, Attention U-Net achieves the highest performance, with Accuracy at 97.92%, Precision at 98.76%, Recall at 98.05%, F1 Score at 97.85%, and MCC at 94.1%, highlighting its superior ability to capture both spatial and contextual features. Overall, these radar plots help monitor the training process, ensuring the models are effectively learning to identify tumor regions rather than overfitting, with Attention U-Net standing out as the most effective in training-based segmentation performance.

**Table 4:** Segmentation metrics comparison with existing models.

Model + loss Function	Dice	IoU	Precision	Recall	F1 Score
U-Net + PIXIE^15^	89.8	81.7	93.0	86.2	89.44
BM-KFO+ (CE + AHD Loss)^16^	74.0	58.73	70.6	79.2	74.0
MBSNet^22^	75.17	69.57	77.0	77.1	77.0
SwinUNet^19^	72.00	56.25	74.0	70.0	72.0
ResNet50 + A-FL^17^	76.9	69.6	94.89	94.1	94.49
UNet^41^	74.64	59.55	72.5	75.2	73.8
AttUNet^42^	70.52	54.47	71.1	72.3	71.7
UNet ++^43^	73.71	58.37	74.2	72.8	73.4
TransUNet^44^	80.00	66.66	86.2	84.3	85.2
ERN101^45^	81.00	68.06	87.5	85.2	86.3
BLG-Net^46^	82.00	69.50	89.4	87.3	88.3
**Attention Unet with (proposed SDHL + BCE) loss function**	**90.16**	**80.78**	**98.76**	**98.05**	**98.57**

The proposed Attention U-Net with SDHL + BCE loss function stands out as the top-performing approach across all metrics is explained in Table 4 among existing methods (89.8%), its IoU (81.7%) and F1 Score (89.44%) fall short of the proposed model. Similarly, models like BLG-Net, ERN101, and TransUNet demonstrate strong performance with F1 scores of 88.3%, 86.3%, and 85.2% respectively, yet they do not match the consistent superiority of the proposed approach across all key indicators.Lower-performing models such as BM-KFO+ (CE + AHD) and SwinUNet exhibit significant weaknesses, particularly in IoU and Dice scores, reflecting limited effectiveness in boundary and region segmentation. While ResNet50 + A-FL achieves high precision (94.89%) and recall (94.1%), it still lags behind the proposed model in terms of overall segmentation quality. The proposed Attention U-Net with SDHL + BCE loss function delivers the best balance of boundary alignment and region accuracy, making it the most reliable and robust choice for medical image segmentation tasks among all compared methods.

### Ablation study

**Table 5:** Ablation study.

Model	Loss Function	Evaluation Metrics				
		Accuracy	Precision	Recall	Dice_Score	IoU
Attention U Net	HDL	74.25	65.53	66.45	64.75	57.61
	BCE	97.37	88.95	85.09	87.72	79.65
	HDL + BCE	99.52	90.74	91.39	89.77	80.47
	SDHL	77.25	68.34	69.71	67.16	60.58
	**Our Proposed Loss(SDHL) + BCE**	**99.71**	**91.06**	**91.85**	**90.16**	**80.78**

The ablation study clearly demonstrates the effectiveness of different loss functions when applied to the Attention U-Net model, with a particular focus on the impact of the proposed SDHL + BCE loss function. Using Hausdorff Distance Loss (HDL) alone yields the lowest performance across all metrics, with an accuracy of 74.25% and a Dice score of just 64.75%, indicating poor segmentation quality and boundary alignment. When Binary Cross-Entropy (BCE) is used alone, there is a significant improvement across all metrics, especially in accuracy (97.37%), precision (88.95%), and Dice score (87.72%), reflecting better region segmentation.CombiningHDL with BCE further enhances performance, achieving a Dice score of 89.77% and accuracy of 99.52%, suggesting that integrating boundary awareness with region-based loss is beneficial. However, replacing HDL with the Sub-Differentiable Hausdorff Loss (SDHL) alone provides moderate improvements over HDL, but still underperforms compared to BCE or the combined losses. The proposed SDHL + BCE loss function achieves the highest performance across all evaluation metrics, including accuracy (99.71%), precision (91.06%), recall (91.85%), Dice score (90.16%), and IoU (80.78%). This highlights its superior ability to balance both region accuracy and boundary alignment, confirming its effectiveness as a robust loss function for medical image segmentation (Table 5).

The computational cost of the proposed Sub-Differentiable Hausdorff Loss (SDHL) integrated segmentation model in terms of trainable parameters, floating-point operations per second (FLOPs), and inference time per image. The analysis demonstrates that the proposed model maintains a balance between accuracy and efficiency, showing only a marginal increase in computational cost compared to standard U-Net, while achieving significantly improved segmentation performance.

- Number of parameters: 12.8 million (proposed model) vs. 11.9 million (U-Net).
- FLOPs: 28.4 GFLOPs per image (512 × 512 resolution).
- Average inference time: 0.42 s per image on NVIDIA RTX A6000 GPU.

These findings confirm that the inclusion of the SDHL loss function introduces minimal additional complexity, making the model computationally feasible for real-time or clinical applications. Based on the results and comparative analyses presented, the final section concludes the study by summarizing the key findings, highlighting the improvements achieved by the proposed method, and outlining potential directions for future work.

The proposed SDHL + BCE loss function consistently achieved superior performance, a few challenging cases revealed minor performance declines. In particular, low-contrast MRI images, irregular tumor boundaries, and small tumor regions caused boundary misclassifications and reduced segmentation precision. We have also outlined possible directions for improvement, such as integrating attention-based feature refinement and transformer driven contextual learning, which could further enhance segmentation accuracy in complex cases.

- The proposed SDHL + BCE loss function demonstrated stable and reliable performance across different segmentation models. However, a few natural limitations related to the study context should be noted. First, the experiments were conducted using a single publicly available MRI dataset. As a result, further investigation is needed to understand how the results may vary across different hospitals, MRI scanners, or multi-center clinical environments.
- In addition, this study primarily used U-Net–based models (U-Net, U-Net+, U-Net++, and Attention U-Net) along with V-Net. Additional research will be useful to explore how the proposed loss function behaves on transformer-based architectures or other advanced segmentation frameworks.
- Finally, the reported results reflect average metric values. A more detailed analysis of performance variation with respect to tumor size, shape, and contrast differences may provide deeper insights in future work. Such evaluations would further strengthen the understanding of the robustness and broader applicability of the proposed loss function.

## Conclusion

In this study, we worked on an MRI brain tumor segmentation dataset and developed a brain tumor segmentation framework to address challenges such as class imbalance, poor sensitivity to small tumors, and the limits of standard loss functions, where SDHL also showed better performance than HDL. To overcome these issues, a novel Sub-Differentiable Hausdorff Loss (SDHL) and a hybrid SDHL + BCE loss function were introduced to enhance spatial accuracy and tumor boundary alignment. Various UNet-derived models, including UNet, UNet+, VNet, UNet++, and Attention UNet, were trained on augmented MRI datasets using the proposed loss functions. Performance evaluation based on metrics such as Accuracy, Precision, Recall, Dice Score, and IoU demonstrated that the model using Attention UNet with SDHL + BCE showed better accuracy and segmentation performance when compared with the other approaches. The results show that the distance-based loss helped the model handle difficult regions better and reduced some segmentation errors.

## Data Availability

The MRI brain tumor segmentation dataset used in this study is publicly available on Kaggle at the following link: [https://www.kaggle.com/datasets/mateuszbuda/lgg-mri-segmentation].
